# Finite Strain Modelling for Multiphase Flow in Dual Scale Porous Media During Resin Infusion Process

**DOI:** 10.1007/s10665-021-10092-9

**Published:** 2021-02-24

**Authors:** Ruoyu Huang

**Affiliations:** Lightweight Manufacturing Centre (LMC), University of Strathclyde, Block E, Westway Business Park, Porterfield Road, Renfrew, PA4 8DJ UK

**Keywords:** Composite manufacturing, Constitutive modelling, Dual scale porosity, Finite strain deformation, Fluid–solid interaction, Multiphase flow, 74E30, 74F10, 76S05, 76T10

## Abstract

Resin infusion is a pressure-gradient-driven composite manufacturing process in which the liquid resin is driven to flow through and fill in the void space of a porous composite preform prior to the heat treatment for resin solidification. It usually is a great challenge to design both the infusion system and the infusion process meeting the manufacturing requirements, especially for large-scale components of aircraft and wind turbine blades. Aiming at addressing the key concerns about flow fronts and air bubble entrapment, the present study proposes a modelling framework of the multiphase flow of resin and air in a dual scale porous medium, i.e. a composite preform. A finite strain formulation is discussed for the fluid–solid interaction during an infusion process. The present study bridges the gap between the microscopic observation and the macroscopic modelling by using the averaging method and first principle method, which sheds new light on the high-fidelity finite element modelling.

## Introduction

In recent years vacuum assisted resin infusion (RI or VARI) becomes an important method for manufacturing the large-scale composite components of structures like airplane wing covers and wind turbine blades [1, 2]. A resin infusion process mainly includes two steps, (1) liquid resin infusion through a dry composite preform and (2) curing (i.e. heat treatment) to transfer the liquid resin into a solid matrix for holding fibres together. From the point of view of mathematical modelling, the former may be considered as a fluid transport process inside a porous medium consisting of a fibre network, the latter is a chemo-thermo-mechanically coupled process with phase changes and the creation of residual stress. The present study is restricted to the step 1, liquid resin infusion process. Therefore, chemical and thermal effects are not discussed explicitly.

**Fig. 1:**
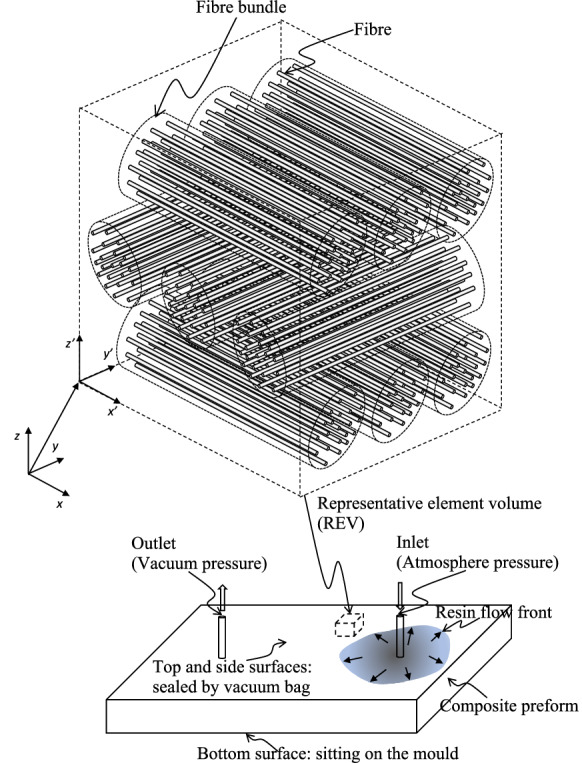
Schematic resin infusion system and the representative element volume (REV) of a composite preform as a dual scale fibre-reinforced porous medium

The schematic illustration of a resin infusion system and a representative element of the composite preform accommodating fluids are shown in Fig. 1. The preform shows a dual scale microstructure, i.e. *fibres* and *bundles* of fibres, which may experience finite strain deformation during a manufacturing process. For non-crimp unidirectional (UD) fabrics, bundles could be stitched together to form plies and a composite preform. This preform is placed such that one side is sitting on the mould and the other side being covered and sealed by a vacuum bag. Inlets under the atmosphere pressure and outlets under the vacuum pressure are connected to either the mould or the vacuum bag for the liquid resin entering or leaving the porous preform driven by the pressure gradient. The aim of liquid resin infusion is to fully fill in the void space of the preform by the resin. In other words, an optimal infusion process should maximise the wetting of a preform with a minimal amount of resin [2]. Thus the design of inlet and outlet system (e.g. locations and the number of outlets/inlets), the assisting flow media to divert flow paths, the time control of opening and closing of inlets and outlets, and the lay-up of composite plies are the key factors to be considered for a successful manufacturing. The challenges are (i) the component scale dry domains formed due to flows taking the *least resistance pathes* and (ii) the bundle and fibre scale air bubbles trapped behind flow fronts. The latter, if cannot be eliminated, will end up with defects in a cured component. This is a potential source of material damage like delamination. Therefore, the key questions are to understand the mechanisms of the formation of dry domains and air bubbles and to address the challenges in the design of infusion system and infusion process. Bearing in mind that this is a fluid–solid interaction process, a fluid’s motion induces changes of the microstructure of a solid skeleton and in turn affects the flow itself.

Darcy’s law has been widely discussed for modelling resin flows in composite manufacturing. The calculation or computation of permeability is one of the key tasks of modelling. Due to the dual scale microstructure of a preform, the liquid resin flow in the void space of a preform is a dual scale flow containing the intra- and inter-bundle flows. Typical length scales of such two types of flow are, $<10\,\upmu$m and $>100\,\upmu$m, respectively [3]. According to the theory of permeability of porous media [4–7], it is clear that the intra-bundle void space has the permeability very different from that of the inter-bundle void space in both magnitude and anisotropy.

Generally, the analytical formulae of Kozeny-Carman [8], Gebart [9] or Berdichevsky [10] may be used to predict the permeability as a function of the volumetric fraction. Based on those formulae, a typical analytical method to estimate the permeability of a dual scale composite preform is to integrate the estimations at different scales together. For example, Gebart [9] proposed a theoretical estimation of the permeability of an idealised unidirectional reinforcement consisting of the regularly ordered, parallel fibres derived from the first principles (i.e. Navier–Stokes equations) both for the flow along and for the flow perpendicular to the fibres. Using Gebart’s intra-bundle result and the additional estimation of permeability of the inter-bundle channels, Lundström [3] suggested the formulation of permeability of dual scale preforms by integrating the intra- and inter-bundle solutions together. Such an analytical estimation usually considers the fluid as a single phase. Thus air bubbles are omitted. Even for a single phase flow, the dual scale microstructure is still highly complicated so that numerical methods have been discussed extensively for predicting the permeability [11–13] and experimental methods have been developed for parameter measurement and model validation [14–17].

The multiphase flow adds more complication to a dual scale system. Considering its relatively lower density and volumetric fraction by comparison to the resin, the air phase can be modelled in various ways subjected to the assumptions applied. The existing models may roughly be classified into two categories: (1) the void space model without explicitly modelling the air phase motion and (2) the air phase motion model.

The void space model naturally refers to the concepts of the *saturated* and *unsaturated* permeabilities [18, 19, 21]. A complicated multiphase flow can be characterised by a phenomenological model of the relative permeabilities with volumetric fractions as the state variables. Such a model generally comes within the scope of Darcy’s law which in principle is limited to a homogeneous and random porous medium [4].

By contrast, the explicit modelling of the air phase motion may provide the micromechanical insight into the mechanisms of void formation and motion. Park et al. [22] and Gangloff et al. [23] reported the detailed hypotheses about the void formation by air entrapment and the air bubble motion aiming at the manufacturing strategy to minimise the void phase. The direct computational fluid dynamics (CFD) modelling may provide the enhanced visualisation for improving the understanding of air-resin interaction [24]. In the circumstance that the air phase is taken into account, mathematical modelling has shown explicitly that Darcy’s law may not cover some key features observed in the dual scale multiphase resin infusion system. Pillai and coworkers [25–27] used the microscopic model and averaging method to demonstrate that the flow flux equation is in a form of Brinkman’s equation, which is consistent with the model proposed by Celle et al. [28].

As the resin and air are two immiscible fluids, it is interesting to understand if capillary forces play important role in the resin infusion. In the well-known Buckley–Leverett theory (see e.g. [29]), the capillary force is ignored so that two immiscible fluids have the same pressure. However, Amico and Lekakou [30, 31] conducted the experimental study and came to the conclusion that the effects of the capillary forces cannot be ignored when the resin pressure is sufficiently low. More precisely, a dimensionless number, either *capillary number*, $Ca^*$, or *bubble mobility*, may be used to characterise the competition between viscous flow and capillary wicking, which may be useful to depict the local flows of resin and air and be important for the removal of voids [22, 23].

Bearing in mind that the resin transports through a solid skeleton composed of fibres and fibre bundles [28, 32], without being glued by a matrix, i.e. the cured resin, fibres and fibre bundles may experience the elastic finite strain deformation during an infusion process. Larsson et al. [34, 35] proposed a model taking the fibre bundle (i.e. the tow including the fibres and intra-tow voids) as a hyperelastic material. Similar to Pillai’s model [25], a bundle is considered as a ‘sink’ to the mass balance of resin in the inter-bundle void space. It is noted that the finite strain deformation is one of the features in the resin infusion process which may not be considered in other dual scale media, e.g. fractured media [33].

In addition to the aforementioned material characterisation and modelling of dual scale porous media, finite element analysis (FEA) has been reported for the component scale modelling of resin flow processes [20, 21, 36]. Commercial software for both material modelling (e.g. Digimat^®^) and process modelling (e.g. ESI-Composites^®^) have achieved success to a certain extent. However, to the author’s best knowledge, especially that of the modelling of industry manufacturing processes of wing covers and wind turbine blades, it is still a great challenge to create a model feasible to cope with the simulation of a manufacturing process of large-scale components with the reasonably useful detail of air bubbles. This is a typical challenge of the time-dependent cross-scale modelling where a component could span up to more than ten metres while the size of individual air bubble may be down to the same scale of fibre diameter. The transient nature of the problem and the pressure-dependency of permeability coupled to the finite strain deformation of the preform during an infusion process make the modelling even more challenging.

In order to address the challenge, the present study proposes a framework of finite strain dual scale fibre-reinforced model explicitly taking into account the multiphase flow of the resin and air. The finite strain modelling of the fibre reinforced soft materials has achieved substantial progress in the last decade [37]. And the image processing and data analysis technologies provided by the advanced imaging software, e.g. Thermo Fisher Avizo^®^ or Materialise^®^, offer the support to construct the more sophisticated microstructure-based constitutive model. Moreover, the increasing data from lab experiments and industry manufacturing practice have not been fully exploited by the mathematical and finite element modelling, let alone predicted by the models. Therefore, it is interesting and important (1) to exploit the advanced finite strain fibre-reinforced model and imaging-based microstructure model for the more sophisticated modelling of resin infusion processes and (2) to construct a *high-fidelity* modelling framework which is able to accommodate the high-precision data of a dual scale flow and in turn predict the high-accuracy detail of interest. For this purpose, the present study adapts the dual porosity concept of fractured media [5] and extended it to a finite strain model of dual scale fibre-reinforced materials. It is assumed that the same fluid (either the resin or the air) in the intra- and inter-bundle void spaces may be considered as two different phases. Such two phases can interchange mass and momentum on their interface. Therefore, there are five phases, i.e. the resin in the intra-bundle void space, the resin in the inter-bundle void space, the air in the intra-bundle void space, the air in the inter-bundle void space, and the solid phase of a preform. Hence, the proposed method may capture some key features of interest, e.g. local flow fronts and defects induced by air bubbles at the different scales. It is also worth emphasising that the proposed framework of finite strain modelling can be extended consistently to cover the curing process by adding the chemo-thermal equations.

The present work is arranged as follows: First, the finite strain kinematical theory is presented to define the dual scale multiphase flow system. Secondly the fundamental balance laws of the individual phase at the microscopic scale are discussed for understanding the motion of each phase and the constitutive law. Thirdly, the macroscopic governing equations of mass balance and momentum balance are obtained by averaging the corresponding microscopic counterparts. The constitutive laws of macroscopic stresses taking into account the capillary forces are also obtained by using the averaging rules. Finally, instead of deducing the macroscopic constitutive laws of fluid fluxes from the macroscopically thermodynamic constraint, the present work discusses the macroscopic fluxes using the averaged momentum balance equations, which would be helpful for clarifying the multiphase flow in dual scale media and shedding new light on the finite element modelling of the manufacturing process of large-scale composite components.

## Kinematics theory

**Fig. 2:**
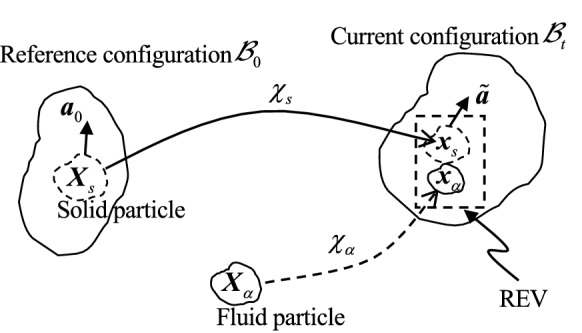
Kinematical relationships of the configurations and particles

We consider an REV in Fig. 1 composed of a deformable solid skeleton and fluids flowing through the void space. Mathematically, the kinematical relations of the different configurations and particles during a motion are shown in Fig. 2.

At a certain initial time, $t_0$, the solid skeleton is represented as a continuum by its reference configuration, ${{\mathcal {B}}}_0$, with material points labelled by ${\varvec{X}_s} \in {{\mathcal {B}}}_0$. At any current time, $t \in \left[ {0, + \infty } \right)$, a mapping $\chi _s :{{\mathcal {B}}}_0 \rightarrow {{\mathcal {B}}}_t$ is a deformation which maps the reference configuration, ${{\mathcal {B}}}_0$, onto the current configuration, ${{\mathcal {B}}}_t$ embedded into the Euclidean space ${\varvec{{\mathbb {R}}}}^3$. The current position of a material point $\varvec{X}_s$ is written as $\varvec{x}_s = \chi _s (\varvec{X}_s,t) \in {{\mathcal {B}}}_t \subset {\varvec{{\mathbb {R}}}}^3$. The deformation gradient is defined as ${\varvec{F}}_s(\varvec{X}_s,t) = {\partial \chi _s}/{\partial \varvec{X}_s}$ with its Jacobian denoted as $J_s = \det \varvec{F}_s > 0$.

The solid skeleton may be characterised by a *dual scale porosity* fibre-reinforced microstructure, i.e. the fibre bundle package in which each bundle is consisting of one family of fibres (see Fig. 1). Let a unit vector, ${\varvec{a}}_0(\varvec{X}_s)$, denote the initial fibre direction of the family of fibres attached to a material point, $\varvec{X}_s$, of a solid particle on ${{\mathcal {B}}}_0$. The current *spatial* fibre direction at the same material point is

1 $$\begin{aligned} \tilde{\varvec{a}}= \varvec{a}/\sqrt{\lambda _{{\varvec{a}}}}, \end{aligned}$$

where the deformed fibre and its stretch are described by

2 $$\begin{aligned} \varvec{a}= {\varvec{F}}_s({\varvec{X}}_s,t)\varvec{a}_0({\varvec{X}}_s) \quad \mathrm{and}\quad \lambda _{{\varvec{a}}}= {\varvec{C}}_s : {\varvec{A}}_0=\mathrm{tr}{\varvec{A}}, \end{aligned}$$

in which ‘:’ denotes the double contraction, ‘$\mathrm tr$’ is the trace operator, the right Cauchy–Green tensor is defined as

3 $$\begin{aligned} {\varvec{C}}_s= {\varvec{F}}_s^\mathrm{{T}}{\varvec{F}}_s, \end{aligned}$$

and ${\varvec{A}}_0={\varvec{a}}_0\otimes {\varvec{a}}_0$ and ${\varvec{A}}={\varvec{a}}\otimes {\varvec{a}}$ are two structural tensors.

For studying the fluid–solid interaction, it is convenient to consider a *spatial* REV defined by a representative domain of the solid skeleton on ${{\mathcal {B}}}_t$ and the associated *current* void space. Porosity, i.e. the volumetric fraction in the REV, as the first order correlation function [38] is used to characterise the void space of a dual scale microstructure. Accordingly, the REV can be characterised by two independent porosities, e.g. the porosity of the total void space, $\phi$, and that of the intra-bundle void space, $\phi _f$. The porosity of inter-bundle void space is denoted $\phi _b$, where $\phi _b=\phi -\phi _f$. The subscripts *b* and *f* stand for the inter-bundle void space surrounding the *bundles* and the intra-bundle void space surrounding the *fibres*, respectively.

**Fig. 3:**
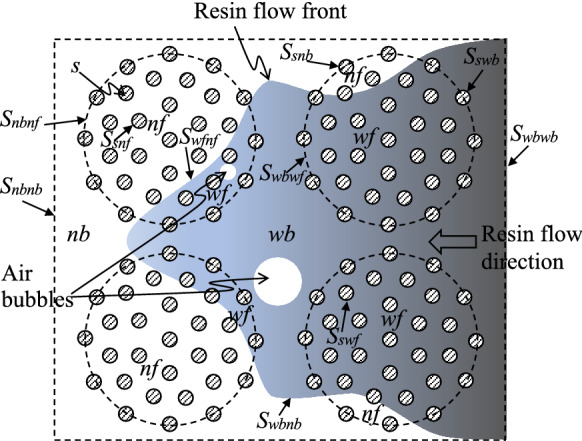
Different phases and their boundaries in a cross section of an REV which include each phase’s own boundary on the surface of the REV and the interfaces between the different phases inside the REV (the shaded area represents the domain wetted by resin)

**Fig. 4:**
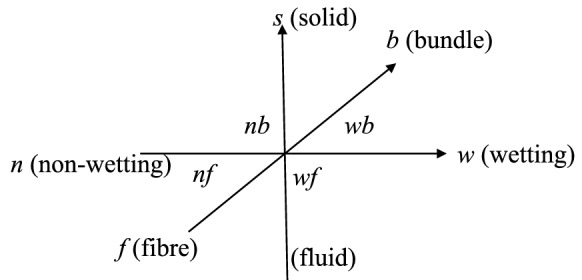
Phase diagram which represents the topological relationships of the different phases in a 3-axes system: (1) the solid/fluid axis, (2) the bundle/fibre axis, and (3) the wetting/non-wetting axis. Noting that there is neither contact between *wf* and *nb* nor between *wb* and *nf*

The void space is initially fully occupied by the air close to the vacuum pressure. Driven by the pressure gradient created by the pressure differences between inlets and outlets (Fig. 1), the liquid resin may gradually enter and occupy part or all of the void space as illustrated in Fig. 3. Therefore, the resin and air are considered as two immiscible fluids and denoted as *w*- and *n*-fluids, respectively. In a conventional way, the *w*-fluid stands for the wetting fluid while *n*-fluid for the non-wetting one. Together, the *w*- and *n*-fluids completely occupy the void space in an REV. For effectively modelling the multiphase flow in the dual scale void space, we refer to a fluid in the intra-bundle void space and to the same fluid occupying the inter-bundle void space as two ‘apparent phases’ [5]. Thus, there are *five* phases in total, denoted *s*, *wb*, *wf*, *nb*, and *nf*, referring to (i) the *solid* phase, (ii) the *w-fluid between the bundles* in the inter-bundle void space, (iii) the *w-fluid between the fibres* in the intra-bundle void space, (iv) the *n-fluid between the bundles* in the inter-bundle void space, and (v) the *n-fluid between the fibres* in the intra-bundle void space, respectively (see Fig. 3 for the geometric relations and Fig. 4 for the topological relations). Hence, we have the relations

4 $$\begin{aligned} \phi =\phi _b+\phi _f, \quad \phi _b=\theta _{wb}+\theta _{nb}, \quad \phi _f=\theta _{wf}+\theta _{nf}, \quad \theta _s=1-\phi , \end{aligned}$$

where $\theta _{\alpha }$ represents the current volumetric fraction of the $\alpha$-phase ($\alpha \in \{s, wb, wf, nb, nf\}$) in an REV. The total volumetric fractions of the *w*- and *n*-fluids read

5 $$\begin{aligned} \theta _w=\theta _{wb}+\theta _{wf} \quad \mathrm{and}\quad \theta _n=\theta _{nb}+\theta _{nf}, \end{aligned}$$

respectively.

Based on the above characterisation of the void space, we can define the kinematics of the fluids in an REV by pulling an $\alpha$-fluid particle located at $\varvec{x}_\alpha \in {\varvec{{\mathbb {R}}}}^3$ in the current void space at time *t* back to its position, ${\varvec{X}}_\alpha \in {\varvec{{\mathbb {R}}}}^3$, at the initial time $t_0$ (Fig. 2). Therefore, the kinematics of the solid and fluids may be defined in a single framework. Let ${\varvec{X}}_\alpha \in {\varvec{{\mathbb {R}}}}^3$ ($\alpha \in \{s, wb, wf, nb, nf\}$) be a material *particle* of the $\alpha$-phase as a continuum. At the current time *t*, the location of a ${\varvec{X}}_\alpha$-particle at a *point* in an REV may be expressed by a *spatial* coordinate vector, $\varvec{x}_\alpha = \chi _\alpha ({{\varvec{X}}_\alpha },t) \in {\varvec{{\mathbb {R}}}}^3$ via a mapping $\chi _\alpha :{\varvec{{\mathbb {R}}}}^3 \rightarrow {\varvec{{\mathbb {R}}}}^3$. Noting that $\chi _\alpha (\varvec{X}_\alpha ,t)=\chi _\beta (\varvec{X}_\beta ,t)$ ($\alpha \ne \beta$) holds only if two particles, $\varvec{X}_\alpha$ and $\varvec{X}_\beta$, overlap on the current interface between the $\alpha$- and the $\beta$-phases.

The material velocity of the $\alpha$-phase is defined as

6 $$\begin{aligned} \varvec{V}_\alpha (\varvec{X}_\alpha ,t)\equiv \dot{\varvec{x}}_\alpha \equiv \frac{{\mathrm{D}}\varvec{x}_\alpha }{\mathrm{D} t}=\frac{\partial {\chi }_\alpha (\varvec{X}_\alpha ,t)}{\partial t}\bigg |_{\varvec{X}_\alpha =\mathrm const}, \end{aligned}$$

where ${\dot{{\phantom{a}}}}\equiv {{\mathrm{D}}}/{\mathrm{D} t}$ is the material derivative. The spatial velocity gradient of the $\alpha$-phase is

7 $$\begin{aligned} {\varvec{L}}_\alpha =\nabla \varvec{V}_\alpha \equiv \frac{\partial \varvec{V}_\alpha }{\partial \varvec{x}_\alpha }, \end{aligned}$$

where $\nabla$ is the spatial gradient operator. It can be proven that ${\varvec{L}}_s=\dot{{\varvec{F}}}_s{\varvec{F}}_s^{-1}$ for the solid phase.

## Microscopic balance equations

Let’s consider a *current* moving material domain of the $\alpha$-phase, $\Omega _\alpha ({\varvec{X}}_\alpha ,t)$, mapped from a reference material domain, $\Omega _\alpha ^0({\varvec{X}}_\alpha )(\equiv \Omega _\alpha ({\varvec{X}}_\alpha ,t_0))$ by $\chi _\alpha :{\varvec{{\mathbb {R}}}}^3 \rightarrow {\varvec{{\mathbb {R}}}}^3$. The surface of $\Omega _\alpha ({\varvec{X}}_\alpha ,t)$, denoted $\Gamma _\alpha ({\varvec{X}}_\alpha ,t)$, is a *material surface* with respect to the $\alpha$-phase moving in a velocity ${\varvec{V}}_\alpha$. There is no mass flux of the $\alpha$-phase passing through the $\Gamma _\alpha$-surface. The *intrinsic* density of the $\alpha$-phase (per unit volume of $\Omega _\alpha$) is denoted $\rho _\alpha$ which generally is a function of pressure and temperature.

### Mass balance of a phase

In the case that there is no mass source (growth) or sink (absorption) in $\Omega _\alpha ({\varvec{X}}_\alpha ,t)$, a mass balance simply reads

8 $$\begin{aligned} \frac{\mathrm{D}}{\mathrm{D} t}\int \limits _{\Omega _\alpha ({\varvec{X}}_\alpha ,t)}\rho _\alpha \,{\mathrm{{d}}U}=0. \end{aligned}$$

The Reynolds transport theorem [5, 39] states that

9 $$\begin{aligned} \frac{\mathrm{D}}{\mathrm{D} t}\int \limits _{\Omega _\alpha ({\varvec{X}}_\alpha ,t)}(*)\,{\mathrm{{d}}U}=\int \limits _{\Omega _\alpha ({\varvec{X}}_\alpha ,t)}{\frac{\partial (*)}{\partial t}\,\mathrm{{d}}U}+\int \limits _{\Gamma _\alpha ({\varvec{X}}_\alpha ,t)}{(*){\varvec{V}}_\alpha \cdot {\varvec{\nu }}_\alpha \,\mathrm{{d}}S}, \end{aligned}$$

in which ${\varvec{\nu }}_\alpha$ is the outer normal vector of the $\Gamma _\alpha$-surface. Thus, by using Gaussian theorem, Eq. (8) becomes

10 $$\begin{aligned} \frac{\mathrm{D}}{\mathrm{D} t}\int \limits _{\Omega _\alpha ({\varvec{X}}_\alpha ,t)}\rho _\alpha \,{\mathrm{{d}}U} =\int \limits _{\Omega _\alpha ({\varvec{X}}_\alpha ,t)}{\frac{\partial \rho _\alpha }{\partial t}+\nabla \cdot (\rho _\alpha {\varvec{V}}_\alpha )\,\mathrm{{d}}U} =\int \limits _{\Omega _\alpha ({\varvec{X}}_\alpha ,t)}{\frac{\mathrm{D\rho _\alpha }}{\mathrm{D} t}+\rho _\alpha \nabla \cdot {\varvec{V}}_\alpha \,\mathrm{{d}}U}, \end{aligned}$$

where $${\mathrm{D(*)}}/{\mathrm{D} t}={\partial (*)}/{\partial t}+{\varvec{V}}_\alpha \cdot \nabla (*)$$.

Since $\Omega _\alpha ^0({\varvec{X}}_\alpha )$ is an arbitrary domain, the local format of the mass balance of an $\alpha$-phase is obtained from Eq. (10) as

11 $$\begin{aligned} \frac{\partial \rho _\alpha }{\partial t}=-\nabla \cdot (\rho _\alpha \varvec{V}_\alpha ) \quad \mathrm{or}\quad \frac{\mathrm{D_\alpha }\rho _\alpha }{\mathrm{D} t} =-\rho _\alpha \nabla \cdot {\varvec{V}}_\alpha \equiv -\rho _\alpha \,\mathrm{tr}\,{{\varvec{L}}}_\alpha . \end{aligned}$$

### Linear momentum balance of a phase

The linear momentum balance of the moving material domain of an $\alpha$-phase, $\Omega _\alpha ({\varvec{X}}_\alpha ,t)$, bounded by the surface, $\Gamma _\alpha ({\varvec{X}}_\alpha ,t)$, reads

12 $$\begin{aligned} \frac{\mathrm{D}}{\mathrm{D} t}\int \limits _{\Omega _\alpha ({\varvec{X}}_\alpha ,t)}\rho _\alpha {\varvec{V}_\alpha }\,\mathrm{{d}U} =\int \limits _{\Gamma _\alpha ({\varvec{X}}_\alpha ,t)}{\varvec{\nu }}_\alpha \cdot {\varvec{\sigma }_\alpha }\,\mathrm{{d}S}, \int \limits _{\Omega _\alpha ({\varvec{X}}_\alpha ,t)}\rho _\alpha {\varvec{f}_\alpha }\,\mathrm{{d}U}, \end{aligned}$$

where ${\varvec{\sigma }}_\alpha$ and $\varvec{f}_\alpha$ are the Cauchy stress tensor and body force vector applied on the $\alpha$-phase, respectively.

Following the similar process to obtain Eq. (11), the local format of the balance of linear momentum is obtained as

13 $$\begin{aligned} \frac{\partial (\rho _\alpha {\varvec{V}_\alpha })}{\partial t}=-\nabla \cdot (\rho {\varvec{V}_\alpha }\otimes {\varvec{V}_\alpha }-\varvec{\sigma }_\alpha )+\rho _\alpha \varvec{f}_\alpha , \end{aligned}$$

where $\rho {\varvec{V}_\alpha }\otimes {\varvec{V}_\alpha }$ and $\varvec{\sigma }_\alpha$ are interpreted as the rates of momentum gained by the advection and diffusive momentum transfers, respectively, $\varvec{f}_\alpha$ represents the rate of supply of momentum by a body force, all terms are per unit volume of the $\alpha$-phase.

By using the mass balance (11), Eq. (13) can be re-expressed in terms of the velocity $\varvec{V}_\alpha$, known as *equation of motion*, as

14 $$\begin{aligned} \rho _\alpha \frac{\partial {\varvec{V}_\alpha }}{\partial t}=-\rho _\alpha {\varvec{V}_\alpha }\cdot \nabla {\varvec{V}_\alpha }+\nabla \cdot \varvec{\sigma }_\alpha +\rho _\alpha \varvec{f}_\alpha , \end{aligned}$$

or in an equivalent form

15 $$\begin{aligned} \frac{\mathrm{D}{\varvec{V}_\alpha }}{\mathrm{D} t}=\frac{1}{\rho _\alpha }\nabla \cdot \varvec{\sigma }_\alpha +\varvec{f}_\alpha . \end{aligned}$$

### Energy balance of a phase

The density of the total energy of the $\alpha$-phase, denoted $e_\alpha$, per unit volume of $\Omega _\alpha ({\varvec{X}}_\alpha ,t)$, is the sum of its thermodynamic internal energy part and kinetic energy part,

16 $$\begin{aligned} e_\alpha =\rho _\alpha I_\alpha +\tfrac{1}{2}\rho _\alpha \parallel {\varvec{V}_\alpha }\parallel ^2, \end{aligned}$$

where $I_\alpha$ is the specific internal energy (internal energy per unit mass), $\parallel {\varvec{*}}\parallel =\sqrt{\varvec{*}\cdot \varvec{*}}$ is the Euclidean norm of a vector.

The energy balance of the $\alpha$-phase states that

17 $$\begin{aligned} \frac{\mathrm{D}}{\mathrm{D} t}\int \limits _{\Omega _\alpha ({\varvec{X}}_\alpha ,t)}e_\alpha \,{\mathrm{{d}}U}= & {} -\int \limits _{\Gamma _\alpha ({\varvec{X}}_\alpha ,t)}{e_\alpha \varvec{V}_\alpha \cdot \varvec{\nu }_\alpha \,\mathrm{{d}}S} +\int \limits _{\Gamma _\alpha ({\varvec{X}}_\alpha ,t)}{\varvec{\nu }_\alpha \cdot \varvec{\sigma }_\alpha \cdot \varvec{V}_\alpha \,\mathrm{{d}}S} \nonumber \\&\quad +\,\int \limits _{\Omega _\alpha ({\varvec{X}}_\alpha ,t)}{\rho _\alpha \varvec{f}_\alpha \cdot \varvec{V}_\alpha \,\mathrm{{d}}U} -\int \limits _{\Gamma _\alpha ({\varvec{X}}_\alpha ,t)}{\varvec{j}_\alpha ^H\cdot \varvec{\nu }_\alpha \,\mathrm{{d}}S} +\int _{\Omega _\alpha ({\varvec{X}}_\alpha ,t)}{\rho _\alpha \Gamma _\alpha ^H\,\mathrm{{d}}U}, \end{aligned}$$

where the various terms on the right hand side (r.h.s) of Eq. (17) contributing to the energy balance are: (i) the advective energy supplied through the $\Gamma _\alpha$-surface in the first integral on the r.h.s, (ii) the work of forces acting on the phase by the surface force, $\varvec{\nu }_\alpha \cdot \varvec{\sigma }_\alpha$, in the second integral, (iii) the work of forces acting on the phase by the body force, $\varvec{f}_\alpha$, per unit mass in the third integral, (iv) the diffusive energy supplied through the $\Gamma _\alpha$-surface by a heat flux, $\varvec{j}_\alpha ^H$, in the fourth integral, and (v) the rate of production of energy within $\Omega _\alpha$ by a heat source, $\Gamma _\alpha ^H$, in the last integral.

The local format of Eq. (17) can be worked out as

18 $$\begin{aligned} \frac{\partial e_\alpha }{\partial t}=-\nabla \cdot (e_\alpha \varvec{V}_\alpha +\varvec{j}_\alpha ^H)+\nabla \cdot (\varvec{\sigma }_\alpha \cdot \varvec{V}_\alpha )+\rho _\alpha \varvec{f}_\alpha \cdot \varvec{V}_\alpha +\rho _\alpha \Gamma _\alpha ^H. \end{aligned}$$

By using the mass balance (11) and linear momentum balance (14), Eq. (18) can be re-expressed in a concise form

19 $$\begin{aligned} \rho _\alpha \frac{\mathrm{D}I_\alpha }{\mathrm{D} t}= \varvec{\sigma }_\alpha :{\varvec{L}}_\alpha -\nabla \cdot \varvec{j}_\alpha ^H +\rho _\alpha \Gamma _\alpha ^H. \end{aligned}$$

For obtaining the constitutive model feasible to FEA, the *Helmholtz free energy* of solid phase, $\psi _s$, and *Gibbs free energy* of an $\alpha$-fluid, $\varphi _\alpha$, are introduced respectively as follows:

20 $$\begin{aligned} \psi _s=I_s-Ts_s \quad \mathrm{and}\quad \varphi _\alpha =I_\alpha -Ts_\alpha +\rho _\alpha ^{-1}P_\alpha \ln J_\alpha \quad \forall \alpha \in \{wb, wf, nb, nf\}, \end{aligned}$$

where *T* is temperature, $s_\alpha$ ($\alpha \in \{s, wb, wf, nb, nf\}$) is the specific entropy (per unit mass) of the $\alpha$-phase. Given the $\alpha$-fluid’s pressure, $p_\alpha$, defined on the current fluid particle at ${\varvec{x}}_\alpha$,

21 $$\begin{aligned} P_\alpha =(1+\ln J_\alpha )^{-1}p_\alpha \end{aligned}$$

is the *pull-back* of $p_\alpha$ from ${\varvec{x}}_\alpha$ to ${\varvec{X}}_\alpha$ in the way as transferring the Cauchy stress to the second Piola–Kirchhoff stress.

By substituting Eq. (20)$_1$ into Eq. (19), the energy balance of the *s*-phase becomes

22 $$\begin{aligned} \rho _s\frac{\mathrm{D}s_s}{\mathrm{D} t} =\frac{1}{T}\left\{ -\rho _s \frac{\mathrm{D}\psi _s}{\mathrm{D} t} +\varvec{\sigma }_s:\varvec{L}_s -T\nabla \cdot (T^{-1}\varvec{j}_s^H) -T^{-1}\varvec{j}_s^H\cdot \nabla T +\rho _s \Gamma _s^H -\rho _s s_s\frac{\mathrm{D}T}{\mathrm{D} t} \right\} . \end{aligned}$$

Similarly, by substituting Eq. (20)$_2$ into Eq. (19) and using the relationships,

23 $$\begin{aligned} \frac{1}{J_\alpha }\frac{\mathrm{D}J_\alpha }{\mathrm{D} t}=-\frac{1}{\rho _\alpha }\frac{\mathrm{D}\rho _\alpha }{\mathrm{D} t}=\mathrm{tr}\;\varvec{L}_\alpha , \end{aligned}$$

the energy balance of an $\alpha$-fluid ($\alpha \in \{ wb, wf, nb, nf\}$) is obtained as

24 $$\begin{aligned} \rho _\alpha \frac{\mathrm{D}s_\alpha }{\mathrm{D} t} =\frac{1}{T}\left\{ -\rho _\alpha \frac{\mathrm{D}\varphi _\alpha }{\mathrm{D} t} +\dot{P}_\alpha \ln J_\alpha +\varvec{\tau }_\alpha :\varvec{L}_\alpha -\nabla \cdot \varvec{j}_\alpha ^H +\rho _\alpha \Gamma _\alpha ^H -\rho _\alpha s_\alpha \frac{\mathrm{D}T}{\mathrm{D} t} \right\} . \end{aligned}$$

Whereby we use the stress decomposition of an $\alpha$-fluid,

25 $$\begin{aligned} {\varvec{\sigma }}_\alpha ={\varvec{\tau }}_\alpha -p_\alpha {\varvec{I}}, \end{aligned}$$

which decomposes the Cauchy stress ${\varvec{\sigma }}_\alpha$ into the viscous stress ${\varvec{\tau }}_\alpha$ and pressure $p_\alpha$.

### Entropy balance and constitutive modelling

#### Entropy balance equation

Equations (22) and (24) are the expressions of the changes of specific entropies derived from the *energy balances*. On the other hand, the general form of an *entropy balance* can be expressed as

26 $$\begin{aligned} {\rho _\alpha \frac{\mathrm{D} s_\alpha }{\mathrm{D} t}} =-\nabla \cdot \varvec{j}_\alpha ^S +\rho _\alpha \Gamma _\alpha ^S \quad \forall \alpha \in \{s, wb, wf, nb, nf\}, \end{aligned}$$

where $\varvec{j}_\alpha ^S$ and $\Gamma _\alpha ^S$ are the diffusive entropy flux and rate of production of entropy per unit mass of an $\alpha$-phase, respectively. The second law of thermodynamics states that the dissipation of an $\alpha$-phase, denoted ${\mathcal {D}}_\alpha$, must satisfy the constraint,

27 $$\begin{aligned} {\mathcal {D}}_\alpha =T\Gamma _\alpha ^S\ge 0 \quad \forall \alpha \in \{s, wb, wf, nb, nf\}. \end{aligned}$$

By comparison of Eq. (26) with Eq. (22), it can be identified that for the solid phase the expressions of $\varvec{j}_\alpha ^S$ and $\Gamma _s^S$ are

28 $$\begin{aligned} \varvec{j}_s^S=T^{-1}\varvec{j}_s^H \end{aligned}$$

and

29 $$\begin{aligned} \Gamma _s^S= \frac{1}{T}\left\{ -\rho _s \frac{\mathrm{D}\psi _s}{\mathrm{D} t} +\varvec{\sigma }_s:\varvec{L}_s - T^{-1}\varvec{j}_s^H\cdot \nabla T +\rho _s \Gamma _s^H -\rho _s s_s\frac{\mathrm{D}T}{\mathrm{D} t} \right\} , \end{aligned}$$

respectively.

Similarly, for an $\alpha$-fluid we may compare Eq. (26) with Eq. (24) and identify that

30 $$\begin{aligned} \varvec{j}_\alpha ^S=T^{-1}\varvec{j}_\alpha ^H \end{aligned}$$

and

31 $$\begin{aligned} \Gamma _\alpha ^S= \frac{1}{T}\left\{ -\rho _\alpha \frac{\mathrm{D}\varphi _\alpha }{\mathrm{D} t} +\dot{P}_\alpha \ln J_\alpha +\varvec{\tau }_\alpha :\varvec{L}_\alpha -T^{-1}\varvec{j}_\alpha ^H\cdot \nabla T +\rho _\alpha \Gamma _\alpha ^H -\rho _\alpha s_\alpha \frac{\mathrm{D}T}{\mathrm{D} t} \right\} . \end{aligned}$$

#### Constitutive model of a solid

In order to further explore the thermodynamics constraints upon the constitutive laws, the free energy functions $\psi _s$ and $\varphi _\alpha$ are specified in the forms as

32 $$\begin{aligned} \psi _s={\hat{\psi }}_s(T,{\varvec{C}}_s;{{\varvec{a}}_0}) \quad \mathrm{and}\quad \varphi _\alpha ={\hat{\varphi }}_\alpha (T,P_\alpha ) \quad \forall \alpha \in \{wb, wf, nb, nf\}. \end{aligned}$$

By substituting Eq. (32)$_1$ into Eq. (29) and applying the inequality (27) ($\alpha =s$), the constitutive constraint upon the solid phase is obtained as

33 $$\begin{aligned} \left\{ \varvec{\sigma }_s-2\rho _s {\varvec{F}}_s\frac{\partial {\hat{\psi }}_s}{\partial {\varvec{C}}_s}{{\varvec{F}}}_s^\mathrm{T}\right\} :{\varvec{D}}_s -T^{-1}\varvec{j}_s^H\cdot \nabla T +\rho _s \Gamma _s^H -\rho _s \left( \frac{\partial {\hat{\psi }}_s}{\partial T}+s_s\right) \frac{\mathrm{D_s}T}{\mathrm{D} t} \ge 0, \end{aligned}$$

where ${\varvec{D}}_s$ is the rate of deformation of the solid skeleton defined by

34 $$\begin{aligned} {\varvec{D}}_s\equiv \frac{1}{2}({\varvec{L}}_s+{\varvec{L}}_s^\mathrm{T})=\frac{1}{2}{{\varvec{F}}}_s^{-\mathrm{T}}\dot{{\varvec{C}}}_s{{\varvec{F}}}_s^{-1}. \end{aligned}$$

The inequality (33) indicates the constitutive laws and constraint,

35 $$\begin{aligned} \varvec{\sigma }_s=2\rho _s {\varvec{F}}_s\frac{\partial {\hat{\psi }}_s}{\partial {\varvec{C}}_s}{{\varvec{F}}}_s^\mathrm{T} ,\quad s_s=-\frac{\partial {\hat{\psi }}_s}{\partial T}, \quad \mathrm{and}\quad -T^{-1}\varvec{j}_s^H\cdot \nabla T \ge 0. \end{aligned}$$

The above inequality in (35) may lead to the Fourier’s law of heat flux, $\varvec{j}_s^H=-{\varvec{k}}_s^H\cdot \nabla T$, where ${\varvec{k}}_s^H$ is the thermal conductivity of the solid phase.

Bearing in mind that the solid skeleton has a dual scale microstructure, i.e. fibres and bundles, it may be more convenient to introduce the constitutive law of the fibre bundle as one family fibres at the microstructure scale and to estimate the macroscopic property over an REV by the homogenisation of a package of the bundles. Thus, the free energy function ${\hat{\psi }}_s$ is proposed to have the form as [40]

36 $$\begin{aligned} {\hat{\psi }}_s(T,{\varvec{C}}_s;{\varvec{a}}_0)={\tilde{\psi }}_s(T, J_s,\tilde{{\varvec{C}}}_s;{{\varvec{A}}_0}) ={\tilde{\psi }}_s^\mathrm{{vol}}(T, J_s) +{\tilde{\psi }}_s^\mathrm{iso}(T,{{\tilde{I}}}_1,{{\tilde{I}}}_2,{{\tilde{I}}}_4,{{\tilde{I}}}_5), \end{aligned}$$

where $\tilde{{\varvec{C}}}_s=J_s^{-2/3}{{\varvec{C}}}_s$, and the invariants are defined as

37 $$\begin{aligned} {{\tilde{I}}}_1=\mathrm{tr}\;\tilde{{\varvec{C}}}_s,\quad {{\tilde{I}}}_2=\frac{1}{2}[\mathrm{tr}^2\;\tilde{{\varvec{C}}}_s-\mathrm{tr}\tilde{{\varvec{C}}}_s^2],\quad {{\tilde{I}}}_4={\varvec{A}}_0:\tilde{{\varvec{C}}}_s,\quad {{\tilde{I}}}_5={\varvec{A}}_0:\tilde{{\varvec{C}}}_s^2. \end{aligned}$$

As an example, a particular neo-Hookean free energy function [40] is adopted here

38 $$\begin{aligned} {\hat{\psi }}_s= & {} {\tilde{\psi }}_s(T, J_s,\tilde{{\varvec{C}}}_s;{\varvec{A}}_0) ={\tilde{\psi }}_s^\mathrm{{vol}}(T, J_s)+{\tilde{\psi }}_s^\mathrm{{iso}}(T,{{\tilde{I}}}_1,{{\tilde{I}}}_4)\nonumber \\= & {} \frac{1}{2}\kappa (J_s-1)^2 +\frac{1}{2}\mu ({{\tilde{I}}}_1-3) +\frac{1}{2}\frac{\kappa _1}{\kappa _2}[\exp (\kappa _2({{\tilde{I}}}_4-1)^2)-1], \end{aligned}$$

where $\kappa$ and $\mu$ are interpreted as the bulk modulus and shear modulus, respectively, of the fibre bundle and $\kappa _1$ and $\kappa _2$ are the parameters of the elastic response of the fibres. The explicit expressions of the stresses and elastic tangent modulus derived from the free energy (38) may be referred to [41].

#### Constitutive model of fluids

In the similar way, by substituting Eq. (32)$_2$ into Eq. (31) and applying the inequality (27) ($\alpha \in \{wb, wf, nb, nf\}$), the constitutive constraint upon an $\alpha$-fluid is obtained as

39 $$\begin{aligned} \left( -\rho _\alpha \frac{\partial {\hat{\varphi }}_\alpha }{\partial P_\alpha }+ \ln J_\alpha \right) \dot{P}_\alpha +\varvec{\tau }_\alpha :\varvec{L}_\alpha -T^{-1}\varvec{j}_\alpha ^H\cdot \nabla T +\rho _\alpha \Gamma _\alpha ^H -\rho _\alpha \left( \frac{\partial {\hat{\varphi }}_\alpha }{\partial T}+s_\alpha \right) \frac{\mathrm{D_\alpha }T}{\mathrm{D} t} \ge 0. \end{aligned}$$

Each term on the left-hand side (l.h.s) of inequality (39) is discussed individually.

First, for the arbitrary $\dot{P}_\alpha$ we obtain

40 $$\begin{aligned} \ln J_\alpha =\rho _\alpha \frac{\partial {\hat{\varphi }}_\alpha }{\partial P_\alpha } \quad \mathrm{or}\quad {\hat{\varphi }}_\alpha =\int \limits ^{P_\alpha }\frac{\ln J_\alpha }{\rho _\alpha } \mathrm{{d}} P_\alpha . \end{aligned}$$

Secondly, let’s consider the *Newtonian fluid* with a constitutive law,

41 $$\begin{aligned} {\varvec{\tau }}_\alpha =\mathcal {C}^\prime :{\varvec{D}}_\alpha , \end{aligned}$$

where $\mathcal {C}^\prime$ denotes the fluid’s viscosity coefficient and ${\varvec{D}}_\alpha =\frac{1}{2}({\varvec{L}}_\alpha ^\mathrm{{T}}+{\varvec{L}}_\alpha )$ is the rate of deformation of the $\alpha$-fluid. In this case, the constraint (39) requires that

42 $$\begin{aligned} \varvec{\tau }_\alpha :\varvec{L}_\alpha ={\varvec{D}}_\alpha :\mathcal {C}^\prime :{\varvec{D}}_\alpha \ge 0, \end{aligned}$$

which means the positive-semi-definite condition of $\mathcal {C}^\prime$. Hence, assuming that the molecular structure of the fluids (resin and air) are statistically isotropic, the coefficient $\mathcal {C}^\prime$ is proposed in a form of an isotropic tensor,

43 $$\begin{aligned} \mathcal {C}^\prime =a^\prime \mathcal {I}+a^{\prime \prime }{{\varvec{I}}}\otimes {{\varvec{I}}}, \end{aligned}$$

where $a^\prime$ and $a^{\prime \prime }$ are two scalars, $\mathcal {I}$ is the fourth-order unit tensor with components $\mathcal {I}_{ijkl}=\delta _{ik}\delta _{jl}+\delta _{il}\delta _{jk}$, in which $\delta _{ij}$ is Kronecker delta.

By substituting Eq. (43) into (41), the constitutive law of a compressible isotropic Newtonian fluid (say, air) reads

44 $$\begin{aligned} {\varvec{\tau }}_\alpha =2\mu _\alpha {\varvec{D}}_\alpha +\lambda _\alpha ^{\prime \prime }(\mathrm{tr}\;{\varvec{D}}_\alpha ){{\varvec{I}}}, \end{aligned}$$

and

45 $$\begin{aligned} p_\alpha =-\frac{1}{3}\mathrm{tr}{{\varvec{\sigma }}_\alpha }+\bigg (\lambda _\alpha ^{\prime \prime }+\frac{2}{3}\mu _\alpha \bigg )\mathrm{tr}\,{\varvec{D}}_\alpha , \end{aligned}$$

where $\mu _\alpha =a^\prime$ is the $\alpha$-fluid’s dynamic viscosity or shear coefficient viscosity while $\lambda _\alpha ^{\prime \prime }+\frac{2}{3}\mu _\alpha$ is called the dilatational viscosity or coefficient of bulk viscosity in which $\lambda _\alpha ^{\prime \prime }=a^{\prime \prime }$ [5]. Substituting either (43) or (44) into the constraint (42) yields

$$\begin{aligned} \varvec{\tau }_\alpha :\varvec{L}_\alpha =2\mu _\alpha \mathrm{tr}\,\bigg (\bigg ({\varvec{D}}_\alpha -\frac{1}{3}(\mathrm{tr}\;{\varvec{D}}_\alpha ){{\varvec{I}}}\bigg )^2\bigg )+\bigg (\lambda _\alpha ^{\prime \prime }+\frac{2}{3}\mu _\alpha \bigg )(\mathrm{tr}\,{\varvec{D}}_\alpha )^2 \ge 0, \end{aligned}$$

which indicates that

$$\begin{aligned} \mu _\alpha =a^\prime \ge 0\quad \mathrm{and}\quad \lambda _\alpha ^{\prime \prime }+\frac{2}{3}\mu _\alpha =a^{\prime \prime }+\frac{2}{3}a^\prime \ge 0 \end{aligned}$$

since the two terms, $\mathrm{tr}(({\varvec{D}}_\alpha -({1}/{3})(\mathrm{tr}\,{\varvec{D}}_\alpha ){{\varvec{I}}})^2)$ and $(\mathrm{tr}\,{\varvec{D}}_\alpha )^2$, are independent from each other and both non-negative.

For an incompressible isotropic Newtonian fluid (e.g. resin), Eq. (44) reduces to

46 $$\begin{aligned} {\varvec{\tau }}_\alpha =2\mu _\alpha {\varvec{D}}_\alpha , \end{aligned}$$

and the mean normal stress,

47 $$\begin{aligned} p_\alpha =-\frac{1}{3}\mathrm{tr}\,{\varvec{\sigma }}_\alpha , \end{aligned}$$

is computed by using the incompressibility condition, $\ln J_\alpha =0$ or $\mathrm{tr}{\varvec{D}}_\alpha =0$, while $p_\alpha$ itself may be treated as a Lagrangian multiplier [42].

Thirdly, similar to (35) of the solid counterpart, inequality (39) indicates

48 $$\begin{aligned} s_\alpha =-\frac{\partial {\hat{\varphi }}_\alpha }{\partial T} \quad \mathrm{and}\quad -T^{-1}\varvec{j}_\alpha ^H\cdot \nabla T\ge 0, \end{aligned}$$

which also leads to the Fourier’s law of heat flux, $\varvec{j}_\alpha ^H=-{\varvec{k}}_\alpha ^H\cdot \nabla T$, where ${\varvec{k}}_\alpha ^H$ is the thermal conductivity of the $\alpha$-fluid.

## Macroscopic model of the multiphase flow in a dual porosity medium

As indicated by the specification of free energy functions in (32), a macroscopic model may take the averaged values of the solid’s motion $\chi _s$ and fluids’ pressures $p_\alpha$ ($\forall \alpha \in \{wb, wf, nb, nf\}$) over an REV as the set of macroscopic degree of freedom (DoF). Accordingly, from the point of view of the variational principle and finite element method, the governing equations for computing a macroscopic system may include: (i) the macroscopic mass balance of each fluid phase, (ii) the macroscopic linear momentum balance of the solid skeleton, (iii) the macroscopic constitutive law of the effective stress acting upon the solid skeleton, and (iv) the macroscopic constitutive laws of the mass- or volume-weighted fluxes of the fluids. One may observe that the macroscopic mass balance, macroscopic linear momentum balance and macroscopic constitutive laws of stresses can be obtained by averaging their microscopic counterparts over an REV, which is shown in this section. However, the macroscopic constitutive law of a fluid flux, e.g. Darcy’s law, does not have an apparent microscopic counterpart. Instead, as an application of the *first principle* method, it can be derived from the averaged linear momentum balance equation of the fluid [5, 25].

### Averaging rules

Let’s consider a spatial point, $\varvec{x} \in {{\mathcal {B}}}_t \subset {\varvec{{\mathbb {R}}}}^3$, and its associated REV, denoted $\mathcal {U}_0({\varvec{x}}) \subset {\varvec{{\mathbb {R}}}}^3$, which is fixed in the Euclidean space. The subdomain inside the REV occupied by an $\alpha$-phase is denoted as $\mathcal {U}_{0\alpha }({\varvec{x}},t) \subset \mathcal {U}_0\subset {\varvec{{\mathbb {R}}}}^3$, which is time-dependent.

In order to differentiate a macroscopic model from a microscopic one, a local coordinate system, ${\varvec{x}}^\prime$, is assigned to the REV for the microscopic description (see Fig. 1). This ${\varvec{x}}^\prime$ coordinate system is locally isomorphic to the global coordinate system, ${\varvec{x}}$, for the macroscopic description. Therefore, the configuration of an $\alpha$-phase in the REV is sufficiently described by $\{{\varvec{x}},{\varvec{x}}^\prime ,t\}\in {{\mathcal {B}}}_t \times \mathcal {U}_{0\alpha }\times {\varvec{{\mathbb {R}}}}^+$.

Given a microscopic tensorial field, ${\varvec{G}}({\varvec{x}}^\prime ,t;{\varvec{x}})$, its averages over $\mathcal {U}_{0\alpha }$ and over $\mathcal {U}_0$ are defined, respectively, as

49 $$\begin{aligned} \overline{{\varvec{G}}}^\alpha ({\varvec{x}},t)\triangleq \frac{1}{U_{0\alpha }({\varvec{x}},t)}\int \limits _{\mathcal {U}_{0\alpha }({\varvec{x}},t)}{{\varvec{G}}({\varvec{x}}^\prime ,t;{\varvec{x}}) \,\mathrm{{d}}U} \end{aligned}$$

and

50 $$\begin{aligned} \overline{{\varvec{G}}}({\varvec{x}},t)\triangleq \frac{1}{U_{0}({\varvec{x}})}\int \limits _{\mathcal {U}_0({\varvec{x}})}{{\varvec{G}}({\varvec{x}}^\prime ,t;{\varvec{x}})\gamma ^\alpha ({\varvec{x}}^\prime ,t;{\varvec{x}}) \,\mathrm{{d}}U} \equiv \theta _\alpha ({\varvec{x}},t)\overline{{\varvec{G}}}^\alpha ({\varvec{x}},t), \end{aligned}$$

where

51 $$\begin{aligned} \gamma ^\alpha ({\varvec{x}}^\prime ,t;{\varvec{x}})=\left\{ \begin{matrix} 1 &{} \quad \mathrm{{if}}\quad {{\varvec{x}}}^\prime \in {\mathcal {U}_{0\alpha }}({\varvec{x}},t),\\ 0&{} \quad \mathrm{{if}}\quad {{\varvec{x}}}^\prime \notin {\mathcal {U}_{0\alpha }}({\varvec{x}},t) \\ \end{matrix} \right. \end{aligned}$$

is the characteristic function of the $\alpha$-phase. The volumetric fraction of the $\alpha$-phase, $\theta _\alpha$, is the ratio of the volume of the $\alpha$-phase, $U_{0\alpha }({\varvec{x}},t)$, to the volume of the REV, $U_{0}({\varvec{x}})$,

52 $$\begin{aligned} \theta _\alpha ({\varvec{x}},t)=\frac{U_{0\alpha }({\varvec{x}},t)}{U_0({\varvec{x}})}. \end{aligned}$$

Consequently, we have the decomposition,

53 $$\begin{aligned} {\varvec{G}}({\varvec{x}}^\prime ,t;{\varvec{x}})=\underbrace{\overline{{\varvec{G}}}^\alpha ({\varvec{x}},t)}_{\mathrm{average} }+\underbrace{{\mathring{{\varvec{G}}}}({\varvec{x}}^\prime ,t;{\varvec{x}})}_{\mathrm{deviation}}, \end{aligned}$$

where ${\mathring{{\varvec{G}}}}$ is the deviation of ${\varvec{G}}$ from $\overline{{\varvec{G}}}^\alpha$.

Some useful averaging rules are summarised here (please refer to [5] for the details of derivation). The *averaging rules of sum* and *of product* of two microscopic tensorial fields, ${\varvec{G}}_1$ and ${\varvec{G}}_2$, state that

54 $$\begin{aligned} \overline{{{\varvec{G}}_1+{\varvec{G}}_2}}^\alpha =\overline{{\varvec{G}}_1}^\alpha +\overline{{\varvec{G}}_2}^\alpha \end{aligned}$$

and

55 $$\begin{aligned} \overline{{{\varvec{G}}_1{\varvec{G}}_2}}^\alpha =\overline{{\varvec{G}}_1}^\alpha \overline{{\varvec{G}}_2}^\alpha +\overline{{\mathring{{\varvec{G}}}_1\mathring{{\varvec{G}}}_2}}^\alpha . \end{aligned}$$

The *averaging rules of time derivative* and *of spatial derivative*, are expressed respectively as56and5758where $\nabla _{{\varvec{x}}^\prime }$ and $\nabla _{{\varvec{x}}}$ are the gradient operators in the ${\varvec{x}}^\prime$ and ${\varvec{x}}$ coordinate systems, respectively, and we adopt the symbol [5],59to represent the surface average over an interface, $\mathcal {S}_{\alpha \beta (\alpha )}({\varvec{x}},t)$, in the REV. The set,

$$\begin{aligned} \beta (\alpha )={\underset{\zeta \in \{wb, wf, nb, nf, s\};\zeta \ne \alpha }{\sum \zeta }}, \end{aligned}$$

symbolically represents the union of *all* of the neighbouring phases of an $\alpha$-phase. Hence $\mathcal {S}_{\alpha \beta (\alpha )}({\varvec{x}},t)$ is the the union of *all* of the interfaces between $\mathcal {U}_{0\alpha }$ and any other phase, $\mathcal {U}_{0\zeta } (\zeta \ne \alpha )$, moving with a local velocity ${\varvec{u}}$. $S_{\alpha \beta (\alpha )}$ is the area of $\mathcal {S}_{\alpha \beta (\alpha )}({\varvec{x}},t)$ while ${\bar{S}}_{\alpha \beta (\alpha )}$ is the specific area (per unit volume of the REV), defined as

60 $$\begin{aligned} {\bar{S}}_{\alpha \beta (\alpha )}=\frac{S_{\alpha \beta (\alpha )}}{U_0}. \end{aligned}$$

### Symbolic expressions of the topological relations between phases

Bearing in mind that $\beta (\alpha )$ refers to the set of *all* of the neighbouring phases surrounding an $\alpha$-phase in the REV, this naturally requires the definition of the topological relations of the different phases as shown in Fig. 4. In order to effectively characterise the topology of a dual scale multiphase system, we introduce the symbolic *phase matrices*, ${\varvec{\alpha }}$ and ${\varvec{\beta }}$, to represent the phases and their complete set of neighbours, respectively. We write

61 $$\begin{aligned} {\varvec{\alpha }}= \begin{bmatrix} wb &{} wf &{}0 \\ nb &{} nf &{}0\\ 0&{}0&{}s\\ \end{bmatrix} \end{aligned}$$

and

62 $$\begin{aligned} {\varvec{\beta }}={\varvec{\beta }}^{f}+{\varvec{\beta }}^{fs}, \end{aligned}$$

where the components, $\beta _{ij}^{f}$, refers to the complete fluid–fluid interface, i.e. the set of all of the neighbouring fluid phases of an $\alpha _{ij}$-fluid $(i,j\in \{1,2\})$ while $\beta _{ij}^{fs}$ the complete fluid–solid interface of any $\alpha _{ij}$-phase $(i,j\in \{1,2,3\})$. The interface between the $\alpha _{ij}$-phase and its complete set of neighbours, $\beta _{ij}$, is denoted as $\mathcal {S}_{\alpha _{ij}\beta _{ij}}$. Hence we have

$$\begin{aligned} \mathcal {S}_{\alpha \beta (\alpha )}\equiv \mathcal {S}_{\alpha _{ij}\beta _{ij}}. \end{aligned}$$

Noting that there is no summation over the subscripts *i* and *j*.

It is proposed that $\beta _{ij}^{f}$ may be expressed as a function of the ${\varvec{\alpha }}$’s components in such a form

63 $$\begin{aligned} \beta _{ij}^f=e_{ik3}\alpha _{kj}+e_{jk3}\alpha _{ik}, \end{aligned}$$

where $e_{ijk}$ is the alternating symbol [42] (or the Levi-Civita symbol). Equivalently an explicit matrix form of ${\varvec{\beta }}^f$ is

64 $$\begin{aligned} {\varvec{\beta }}^f= \begin{bmatrix} wf+nb &{} -wb+nf &{}0 \\ -wb+nf &{} -wf-nb &{}0\\ 0&{}0&{}0\\ \end{bmatrix}, \end{aligned}$$

which apperantly can be decomposed into

65 $$\begin{aligned} {\varvec{\beta }}^f={\varvec{\beta }}^\prime +{\varvec{\beta }}^{\prime \prime }, \end{aligned}$$

where

66 $$\begin{aligned} {\varvec{\beta }}^\prime = \begin{bmatrix} wf+nb &{} nf &{}0 \\ nf &{} 0 &{}0\\ 0&{}0&{}0\\ \end{bmatrix} \quad \mathrm{and}\quad {\varvec{\beta }}^{\prime \prime } = \begin{bmatrix} 0 &{} -wb &{}0 \\ -wb &{} -wf-nb &{}0\\ 0&{}0&{}0\\ \end{bmatrix}. \end{aligned}$$

It is clear that either $\cup _{i,j\in 1,2}{\mathcal {S}}_{\alpha _{ij}\beta _{ij}^\prime }$ or $\cup _{i,j\in 1,2}{\mathcal {S}}_{\alpha _{ij}\beta _{ij}^{\prime \prime }}$ is a complete set of the fluid–fluid interfaces in the REV but with opposite normal directions. This explains the meaning of the negative sign in ${\varvec{\beta }}^f$.

The matrix ${\varvec{\beta }}^\prime$ can be further decomposed into

67 $$\begin{aligned} {\varvec{\beta }}^\prime ={\varvec{\beta }}^{c}+{\varvec{\beta }}^{h}, \end{aligned}$$

where

68 $$\begin{aligned} {\varvec{\beta }}^c= \begin{bmatrix} nb &{} nf &{}0 \\ 0 &{} 0 &{}0\\ 0&{}0&{}0\\ \end{bmatrix} \quad \mathrm{and}\quad {\varvec{\beta }}^h = \begin{bmatrix} wf &{} 0&{}0 \\ nf &{} 0 &{}0\\ 0&{}0&{}0\\ \end{bmatrix}. \end{aligned}$$

Hence the set, $\cup _{i,j\in 1,2}{\mathcal {S}}_{\alpha _{ij}\beta _{ij}^c}$, is the complete set of the interfaces between two immiscible fluids (*w* and *n*). On the other hand, ${\mathcal {S}}_{\alpha _{ij}\beta _{ij}^h}$ represents an interface between two phases which are the same fluid (either *w* or *n*) occupying the intra- and inter-bundle void spaces, separately. In other words, ${\mathcal {S}}_{\alpha _{ij}\beta _{ij}^c}$ is an interface between two materials while ${\mathcal {S}}_{\alpha _{ij}\beta _{ij}^h}$ an interface between two scales.

The other form of decomposition of ${\varvec{\beta }}^f$ is

69 $$\begin{aligned} {\varvec{\beta }}^f={\varvec{\beta }}^m+{\varvec{\beta }}^u, \end{aligned}$$

where

70 $$\begin{aligned} {\varvec{\beta }}^m= \begin{bmatrix} wf &{} -wb &{}0 \\ nf &{} -nb &{}0\\ 0&{}0&{}0\\ \end{bmatrix} \quad \mathrm{and}\quad {\varvec{\beta }}^u = \begin{bmatrix} nb &{} nf &{}0 \\ -wb &{} -wf &{}0\\ 0&{}0&{}0\\ \end{bmatrix} \end{aligned}$$

are the miscible and immiscible (unmixable) neighbours of ${\varvec{\alpha }}$, respectively. This complete the discussion about ${\varvec{\beta }}^f$ in Eq. (62).

The remaining term on the r.h.s of (62), ${\varvec{\beta }}^{fs}$, is written as

71 $$\begin{aligned} {\varvec{\beta }}^{fs}= \begin{bmatrix} s &{} s &{}0 \\ s &{} s &{}0\\ 0&{}0&{}-wb-wf-nb-nf\\ \end{bmatrix}, \end{aligned}$$

which does not contribute to mass balance since any fluid–solid surface is a material surface with respect to fluid mass given that there is neither absorption nor dissolution.

It is useful to introduce two matrices,

72 $$\begin{aligned} \tilde{{\varvec{\alpha }}} ={\varvec{\alpha }}+{\varvec{\beta }}^m \quad \mathrm{and}\quad \tilde{{\varvec{\beta }}}={\varvec{\beta }}^{fs}+{\varvec{\beta }}^u. \end{aligned}$$

The former represent the set of $\alpha _{ij}$-phase’s identical fluid (either *w* or *n*) occupying the intra- and inter-bundle void spaces separately while the latter the set of $\alpha _{ij}$-phase’s neighbouring immiscible fluid phase and solid phase. Therefore, ${\mathcal {S}}_{\alpha _{ij}{\tilde{\alpha }}_{ij}}$ is *not* a material surface while ${\mathcal {S}}_{\alpha _{ij}{\tilde{\beta }}_{ij}}$ is such a one.

In order to establish the connection between two index systems, i.e. $\alpha$ and ${\varvec{\alpha }}$, through out the present work the identity,

$$\begin{aligned}\alpha \equiv \alpha _{ij},\end{aligned}$$

holds wherever referring to either $\alpha$ or $\alpha _{ij}$. By comparison to $\beta (\alpha )$, ${\varvec{\beta }}$ and its various decompositions provide a more convenient and clear description about the topological relations.

### Macroscopic mass balance

By using the averaging rules, the averaging of the microscopic mass balance equation (11) yields the macroscopic mass balance equation of an $\alpha$-phase,73Using the proposed phase matrices, Eq. (73) may be re-expressed in a physically equivalent form as74where all material surfaces satisfying $\varvec{\nu }_\alpha \cdot ({\varvec{V}_\alpha }-{\varvec{u}})=0$ are omitted from the surface integral term. The remaining surface integral, , represents the mass exchange of an identical fluid (resin or air) between the intra- and inter-bundle void spaces. Thus it may be interpreted as a ‘sink’ on one side of the ${\mathcal {S}}_{\alpha _{ij}\beta _{ij}^m}$-interface and a ‘source’ on the opposite side.

### Macroscopic linear momentum balance

Averaging the microscopic linear momentum balance equation (13) with the help of the averaging rules, we obtain75To combine with the mass balance equation (73), Eq. (75) becomes76Whereby $\overline{\mathring{\rho }_\alpha {\mathring{\varvec{V}}_\alpha }}^\alpha$ and $\overline{\mathring{(\rho _\alpha {\varvec{V}_\alpha })}\otimes \mathring{\varvec{V}}_\alpha }^{\alpha }$ are the mass and momentum dispersive fluxes, respectively. As one may be aware that $\overline{\mathring{(\rho _\alpha {\varvec{V}_\alpha })}\otimes \mathring{\varvec{V}}_\alpha }^{\alpha }$ is in a form similar to the so-called *Reynolds stress* in the Reynolds-averaged Navier–Stokes (RANS) equations of turbulence theory, the dispersive flux need the additional constitutive law and other equations for the closure of model. As shown by Bear and Bachmat [5], a non-advective flux, including the diffusive and dispersive fluxes, may be grouped with an advective flux into a single Darcy’s law in a phenomenological model. Therefore, the present study focuses on advective fluxes and leaves dispersive fluxes to be discussed elsewhere.

Thus, if eliminating the material surfaces by using Eqs. (62) and (69), and assuming that the mass and momentum dispersive fluxes are much smaller than their advective counterparts, $\overline{\rho _\alpha }^\alpha \overline{{\varvec{V}_\alpha }}^\alpha$ and $\overline{\rho _\alpha {\varvec{V}_\alpha }}^\alpha \otimes \overline{{\varvec{V}_\alpha }}^{\alpha }$, Eq. (76) reduces to such a form77where the macroscopic material derivative, ${\mathrm{D}_\mathrm{m}}/{{\mathrm{D}} t}$, is defined as ${\mathrm{D}_\mathrm{m}(*)}/{\mathrm{D} t}={\partial (*)}/{\partial t}+\overline{{\varvec{V}_\alpha }}^\alpha \cdot \nabla _{{\varvec{x}}}(*)$.

### Macroscopic constitutive model of stresses in a dual porosity medium

Equation (77) indicates the momentum exchanges between the different phases, which is one of the features of a mixture system. Therefore, rather than simply averaging the constitutive laws of each and every phase, the interactions between phases need to be considered for the derivation of a macroscopic constitutive model. This is demonstrated in the derivation of the effective stress of solid skeleton and that of the fluids’ macroscopic fluxes, respectively.

#### Total stress

Summing Eq. (77) over all $\alpha$-phases yields (bearing in mind that $\alpha \equiv \alpha _{ij}$)78where the symbol, $[*]_{\alpha _{ij},\beta _{ij}}$, denotes the jump of ‘$*$’ across $\mathcal {S}_{\alpha _{ij}\beta _{ij}}$ from the $\alpha _{ij}$-phase to the $\beta _{ij}$ one, the total stress is defined as a volumetric averaged stress as

79 $$\begin{aligned} \overline{{{\varvec{\sigma }}}} =\sum _{\alpha _{ij}}\theta _{\alpha _{ij}}\overline{{{\varvec{\sigma }}}_{\alpha _{ij}}}^{\alpha _{ij}} =\sum _{\alpha _{ij}}\overline{{{\varvec{\sigma }}}_{\alpha _{ij}}}, \end{aligned}$$

and the total body force per unit volume of a porous medium is

80 $$\begin{aligned} \overline{\rho \varvec{f}} =\sum _{\alpha _{ij}}\theta _{\alpha _{ij}}\overline{\rho _{\alpha _{ij}}\varvec{f}_{\alpha _{ij}}}^{\alpha _{ij}} =\sum _{\alpha _{ij}}\overline{\rho _{\alpha _{ij}}\varvec{f}_{\alpha _{ij}}}. \end{aligned}$$

Whereby we use (i) the fact that ${\mathcal {S}}_{\alpha _{ij}\beta _{ij}^c}\cup {\mathcal {S}}_{\alpha _{ij}\beta _{ij}^{fs}}$ is a material surface, (ii) the continuity of density and velocity on ${\mathcal {S}}_{\alpha _{ij}\beta _{ij}^h}$, (iii) the continuity of traction on the ${\mathcal {S}}_{\alpha _{ij}\beta _{ij}^h}\cup {\mathcal {S}}_{\alpha _{ij}\beta _{ij}^{fs}}$ surface, i.e.

$$\begin{aligned} \varvec{\nu }_{\alpha _{ij}}\cdot [{{\varvec{\sigma }}}]_{\alpha _{ij},\beta _{ij}^h+\beta _{ij}^{fs}}={\varvec{0}} \quad i,j\in \{1,2\}, \end{aligned}$$

and (iv) the assumption that the surface tension phenomena at the fluid–solid interface, ${\mathcal {S}}_{\alpha _{ij}\beta _{ij}^{fs}}$, is negligible.

Equation (78) indicates the momentum exchanges on (i) the solid–fluid interface, ${\mathcal {S}}_{\alpha _{ij}\beta _{ij}^{fs}}$, which requests to differentiate the effective stress from the total stress; (ii) the interface between the immiscible fluids, ${\mathcal {S}}_{\alpha _{ij}\beta _{ij}^{c}}$, which leads to the concept of capillary pressure; and (iii) the interface between the intra- and inter-bundle void spaces, ${\mathcal {S}}_{\alpha _{ij}\beta _{ij}^{h}}$, which contributes to the coupling effects between two scales.

One may observe that the last term on the r.h.s of Eq. (78) plays a similar role as that of a body force in the total momentum balance. It may be interpreted as the *rate of supply of momentum* by the difference in the macroscopic velocity, $\overline{\varvec{V}}$, between the (same) fluids in the intra-bundle and in the inter-bundle void spaces. This is consistent to some other researchers’ postulation of a ‘sink’ term (see e.g. [25]) in the momentum balance of an inter-bundle fluid where the intra-bundle void space plays the role of a sink through the absorption of resin from the inter-bundle void space. However, in the present study there is no need to identify if it is a source or a sink as the formulation allows either direction of momentum exchange. And it is worth noting that such momentum exchange is governed by the mass balance so this term does not request an additional constitutive model.

#### Capillary pressure

The momentum exchange on the ${\mathcal {S}}_{\alpha _{ij}\beta _{ij}^{c}}$-interface between the immiscible fluids is shown in the first surface integral on the r.h.s of Eq. (78). The continuity of momentum transfer [5] on such a material surface reads

81 $$\begin{aligned} \varvec{\nu }_{\alpha _{ij}}\cdot [{{\varvec{\sigma }}}]_{\alpha _{ij},\beta _{ij}^c} =\frac{2}{r^*}\gamma _{\alpha \beta ^c}\varvec{\nu }_{\alpha _{ij}} -|\nabla _{{{\varvec{x}}}^\prime }\gamma _{\alpha \beta ^c}|{\varvec{t}}, \end{aligned}$$

where $\gamma _{\alpha \beta ^c}$ is the surface tension between the ${\varvec{\alpha }}$ and ${\varvec{\beta }}^c$ fluids, $r^*$ defined by ${1}/{r^*}=({1}/{2})(({1}/{r^\prime })+({1}/{r^{\prime \prime }}))$ is the mean radius of curvature of the $\mathcal {S}_{{\alpha _{ij}}\beta _{ij}^c}$-interface given two principal radii of curvature, $r^{\prime }$ and $r^{\prime \prime }$, and ${{\varvec{t}}}={\nabla _{{{\varvec{x}}}^\prime }\gamma _{\alpha \beta ^c}}/ {|\nabla _{{{\varvec{x}}}^\prime }\gamma _{\alpha \beta ^c}|}$ is a tangential vector on the interface.

Equation (81) represents the force balance on the $\mathcal {S}_{{\alpha _{ij}}\beta _{ij}^c}$-interface which may be decomposed into the force balances in the normal direction ${\varvec{\nu }}_{\alpha _{ij}}$ and tangent direction ${\varvec{t}}$, respectively, as

82 $$\begin{aligned} \varvec{\nu }_{\alpha _{ij}}\cdot [{{\varvec{\tau }}-p{\varvec{I}}}]_{\alpha _{ij},\beta _{ij}^c}\cdot \varvec{\nu }_{\alpha _{ij}} =\frac{2}{r^*}\gamma _{\alpha \beta ^c} \end{aligned}$$

and

83 $$\begin{aligned} \varvec{\nu }_{\alpha _{ij}}\cdot [{{\varvec{\tau }}}]_{\alpha _{ij},\beta _{ij}^c}\cdot {\varvec{t}} = -|\nabla _{{{\varvec{x}}}^\prime }\gamma _{\alpha \beta ^c}| \equiv -\nabla _{{{\varvec{x}}}^\prime }\gamma _{\alpha \beta ^c}\cdot {\varvec{t}}. \end{aligned}$$

If the viscous stress ${\varvec{\tau }}$ and force balance in tangential direction are negligible, Eq. (81) (and (82) as well) reduces to the so-called *Laplace formula* which defines the microscopic capillary pressure,

84 $$\begin{aligned} -[p]_{\alpha _{ij},\beta _{ij}^c} =\frac{2}{r^*}\gamma _{\alpha \beta ^c}. \end{aligned}$$

Hence, the difference in radius between the air bubble in the (intra-bundle) *f*-void space and that in the (inter-bundle) *b*-void space indicates the difference in capillary pressure.

Accordingly, the macroscopic capillary pressure is defined as

85 $$\begin{aligned} p_{\alpha _{ij}\beta _{ij}^c}^c=-\bigg ({\overline{p_{\alpha _{ij}}}}^{\alpha _{ij}}-{\overline{p_{\beta _{ij}^c}}}^{\beta _{ij}^c}\bigg ), \end{aligned}$$

which can contribute to the formulation of the effective stress of solid skeleton as shown below. To be consistent with the microscopic model (84), the phenomenological model of the macroscopic capillary pressure generally is a function of the fluids’ volumetric fractions and surface tension [4, 5],

86 $$\begin{aligned} p_{\alpha _{ij}\beta _{ij}^c}^c={\hat{p}}_{\alpha _{ij}\beta _{ij}^c}^c(\theta _{\alpha _{ij}},\theta _{\beta _{ij}^c},\gamma _{\alpha \beta ^c}), \end{aligned}$$

which can be measured by experimental methods.

#### Effective stress of a solid skeleton

By using the stress decomposition (25) for a fluid, the total stress may be expressed as

87 $$\begin{aligned} \overline{{\varvec{\sigma }}}=\overline{{\varvec{\sigma }}_s} +\sum _{\alpha _{ij}(i,j\in \{1,2\})}\overline{{\varvec{\tau }}_{\alpha _{ij}}} -\left( \sum _{\alpha _{ij}(i,j\in \{1,2\})}\overline{p_{\alpha _{ij}}}\right) \varvec{I}. \end{aligned}$$

Assuming that the viscous stress ${\varvec{\tau }}_{\alpha _{ij}}$ is negligible, Eq. (87) reduces to

88 $$\begin{aligned} \overline{{\varvec{\sigma }}} =\overline{{\varvec{\sigma }}_s} -\overline{p_v}\varvec{I} \equiv \overline{{\varvec{\sigma }}_s^\prime } -\overline{p_v}^v\varvec{I}, \end{aligned}$$

where the *effective stress* [5, 43, 44] is

89 $$\begin{aligned} \overline{{\varvec{\sigma }}_s^\prime } =(1-\phi )(\overline{{\varvec{\sigma }}_s}^s +\overline{p_v}^v\varvec{I}), \end{aligned}$$

and the averaged pressures of all fluids as a whole over the REV and over the whole void space in the REV are defined, respectively, as

90 $$\begin{aligned} \overline{p_v}= \sum _{\alpha _{ij}(i,j\in \{1,2\})}\overline{p_{\alpha _{ij}}} \quad \mathrm{and}\quad \overline{p_v}^v= \frac{1}{\phi }\overline{p_v}. \end{aligned}$$

The subscript ‘*v*’ refers to the whole void space in the REV.

The macroscopic capillary forces defined in (85) may be explicitly introduced into the expression of $\overline{p_v}^v$ as

91 $$\begin{aligned} \overline{p_v}^v= \frac{1}{\phi }(\phi _b\overline{p_{wb}}^{wb}+\theta _{nb}{p_b^c}+\phi _f\overline{p_{wf}}^{wf}+\theta _{nf}{p_f^c}) \equiv \frac{1}{\phi }(\phi \overline{p_{w}}^{w}+\theta _{n}{p_v^c}), \end{aligned}$$

where the macroscopic capillary pressures in the *b*- and *f*-void spaces, respectively, are

92 $$\begin{aligned} {p_b^c} \equiv p_{wbnb}^c = \overline{p_{nb}}^{nb}-\overline{p_{wb}}^{wb} \quad \mathrm{and}\quad {p_f^c} \equiv p_{wfnf}^c = \overline{p_{nf}}^{nf}-\overline{p_{wf}}^{wf}, \end{aligned}$$

and the averaged pressure of the *w*-fluid (i.e. resin) and averaged capillary pressure of the whole void space are obtained as

93 $$\begin{aligned} \overline{p_{w}}^{w}= \frac{1}{\phi }(\phi _b\overline{p_{wb}}^{wb}+\phi _f\overline{p_{wf}}^{wf})\quad \mathrm{and}\quad {p_v^c}= \frac{1}{\theta _n}(\theta _{nb}{p_b^c}+\theta _{nf}{p_f^c}). \end{aligned}$$

Given the constitutive models of the macroscopic capillary pressures, $p_b^c$ and $p_f^c$ (or $p_v^c$), as the functions of the fluids’ volumetric fractions [4, 5, 45], Eq. (91) indicates that one may choose the macroscopic pressure of the *w*-fluid (resin), $\overline{p_{w}}^{w}$, as the DoF in modelling. This is suitable for FEA and experimental tests as the resin’s pressure at the locations of interest can be measured by using pressure gauges during an infusion process.

#### Macroscopic stresses of individual phases and homogenisation methods

With the capillary pressure, effective stresses, and cross-scale momentum source/sink terms defined, the momentum exchanges in a dual scale multiphase system are clarified. The remaining work to complete the macroscopic constitutive model of stresses is the homogenisation of each phase’s stresses.

The macroscopic stresses of each phase may be obtained from averaging the microscopic constitutive laws of the solid, (35),

94 $$\begin{aligned} \overline{\varvec{\sigma }_s}=2\overline{\rho _s {\varvec{F}}_s\frac{\partial {\hat{\psi }}_s}{\partial {\varvec{C}}_s}{{\varvec{F}}}_s^\mathrm{T}}, \end{aligned}$$

and that of a fluid, (41),

95 $$\begin{aligned} \overline{{\varvec{\tau }}_\alpha }=\overline{\mathcal {C}^\prime :{\varvec{D}}_\alpha }, \quad \forall \alpha \in \{wb,wf,nb,nf\}. \end{aligned}$$

For a compressible fluid, averaging Eq. (40) yields

96 $$\begin{aligned} \overline{\ln J_\alpha }=\overline{\rho _\alpha \frac{\partial {\hat{\varphi }}_\alpha }{\partial P_\alpha }}. \end{aligned}$$

There are different homogenisation methods in literature to obtain the averaged constitutive laws. Roughly those methods were classified into three categories, the Voigt method (assuming an uniform strain in an REV), the Reuss method (assuming an uniform stress in an REV) and the energy equivalency method, e.g. the self-consistent method [38]. In FEA, one may only need to consider the constitutive laws at the discrete Gaussian points of each element [39]. In other words, the averaging equation (49) is computed at the discrete Gaussian point, say ${\varvec{x}}= {\varvec{x}}_\mathrm{Gaussian}$. If $\chi _s$ and $p_\alpha$ ($\alpha \in \{wb,wf,nb,nf\}$) are taken as the DoF of the finite element system, the Voigt method supposes that the gradients of the DoF are uniform in the REV of each (macroscopic) Gaussian point, ${\varvec{x}}_\mathrm{Gaussian}$. This means that strain measure ${\varvec{F}}_s$ is *assumed* uniform in the solid phase while $p_\alpha$ varies monotonously within its domain in the void space of the REV, i.e.

97 $$\begin{aligned} \nabla _{{\varvec{x}}^\prime }^2p_\alpha =0\quad \mathrm{in}\quad {\mathcal {U}}_{0\alpha }. \end{aligned}$$

Such assumed fields for homogenisation are consistent with the finite element’s convergence criterion, e.g. the patch test [39].

## Macroscopic fluxes in dual porosity media

As aforementioned, the macroscopic constitutive law of the mass- or volume-weighted flux of a fluid phase in a porous medium may be derived directly from the thermodynamical constraint, objectiveness, and material symmetries of the specific material at the macroscopic scale [44]. By contrast, taking the averaged momentum balance as the first principle, the micromechanical method directly estimates the macroscopic flux in an REV and consequently provides the constitutive law of the fluid’s flux, e.g. Darcy’s law or its enhanced formats, and the corresponding material property of the porous medium, e.g. permeability [3, 9]. Aiming at understanding the complicated resin infusion process and shedding new lights on a high-fidelity FEA, the present study adapts and extends the micromechanical method proposed in [5] to the finite strain dual scale multiphase system discussed above.

In the view of averaged spatial derivative (58), stress decomposition (25) and the specification of body force as gravity, ${\varvec{f}}=-g\nabla _{{\varvec{x}}^\prime }z^\prime ,$ Eq. (77) can be re-written as98Noting that the l.h.s of above equation is in terms of macroscopic variables defined at ${\varvec{x}}$ while the r.h.s in terms of microscopic ones defined in an REV. Equation (98) is taken as the first principle for estimating the macroscopic fluxes at ${\varvec{x}}$. For this purpose, first, each term on the r.h.s need to be expressed in terms of the macroscopic variables, e.g. $\overline{\varvec{V}_\alpha }^\alpha$ and $\overline{p_\alpha }^\alpha$, to transfer (98) into a fully macroscopic equation. Secondly, a reduced format of (98) is used to obtained the first order formulation of the macroscopic fluxes in a closed form which is consistent to Darcy’s law. Thirdly, the enhanced formulations, e.g. the quadratic formulation and Brinkman-type formulation, are also presented for further exploring Eq. (98).

### Averaging microscopic momentum balance equation in terms of macroscopic variables

#### Averaging the pressure gradient and gravity terms in Eq. (98)

The pressure gradient term on the r.h.s of (98) reads

99 $$\begin{aligned} \theta _\alpha \overline{\nabla _{{\varvec{x}}^\prime }p_\alpha }^\alpha \equiv \overline{\nabla _{{\varvec{x}}^\prime }p_\alpha } =\frac{1}{U_{0}}\int \limits _{\mathcal {U}_{0\alpha }}{\nabla _{{\varvec{x}}^\prime }p_\alpha \,\mathrm{{d}}U} =\frac{1}{U_{0}}\int \limits _{\mathcal {S}_{0\alpha }}{p_\alpha {\varvec{\nu }}_\alpha \,\mathrm{{d}}S}, \end{aligned}$$

where

$$\begin{aligned} \mathcal {S}_{0\alpha }=\mathcal {S}_{\alpha \alpha }\cup \mathcal {S}_{\alpha _{ij}\beta _{ij}}=\mathcal {S}_{\alpha _{ij}{\tilde{\alpha }}_{ij}}\cup \mathcal {S}_{\alpha _{ij}{\tilde{\beta }}_{ij}} \end{aligned}$$

is the complete surface of ${\mathcal {U}}_{0\alpha }$. By using the homogenisation assumption (97), it can be proven that (see Appendix A)

100 $$\begin{aligned} \overline{\nabla _{{\varvec{x}}^\prime }p_\alpha }^\alpha ={\nabla _{{{\varvec{x}}}} \overline{p_\alpha }^\alpha }\cdot {\varvec{T}}_\alpha ^* +\frac{1}{\theta _\alpha U_{0}}\int \limits _{\mathcal {S}_{\alpha _{ij}{\tilde{\beta }}_{ij}}}{(\varvec{\nu }_\alpha \cdot \nabla _{{{\varvec{x}}}^\prime } p_\alpha )\mathring{{\varvec{x}}}^\prime \,\mathrm{{d}}S}, \end{aligned}$$

where the *tortuosity* of the $\alpha$-fluid is

101 $$\begin{aligned} {\varvec{T}}_\alpha ^* =\frac{1}{\theta _\alpha U_{0}}\int \limits _{\mathcal {S}_{\alpha _{ij}{\tilde{\alpha }}_{ij}}}{\varvec{\nu }_\alpha \otimes \mathring{{\varvec{x}}}^\prime \,\mathrm{{d}}S}. \end{aligned}$$

As the resin generally moves slow enough in the preform (except the vicinity of inlet points perhaps) under the proper control in a well-designed infusion system, the resin flow is always at a very small Reynolds number ($Re\ll 1$) [23, 28]. Thus, one may assume that in the vicinity of $\mathcal {S}_{\alpha _{ij}{\tilde{\beta }}_{ij}}$, the normal components of both the inertial force and the viscous resistance to the flow are negligible by comparison to the normal component of pressure gradient and gravity, i.e.

102 $$\begin{aligned} \left| \varvec{\nu }_\alpha \cdot \left( {\rho _\alpha }\frac{\mathrm{D_\alpha }{\varvec{V}_\alpha }}{\mathrm{D} t} -\nabla _{{\varvec{x}}^\prime }\cdot {\varvec{\tau }}_\alpha \right) \right| \ll \left| \varvec{\nu }_\alpha \cdot \left( -\nabla _{{\varvec{x}}^\prime }p_\alpha -\rho _\alpha g\nabla _{{\varvec{x}}^\prime }z^\prime \right) \right| , \end{aligned}$$

as indicated by Eq. (15). Thus, Eq. (15) projected in $\varvec{\nu }_\alpha$ direction reduces to

103 $$\begin{aligned} \varvec{\nu }_\alpha \cdot \nabla _{{\varvec{x}}^\prime }p_\alpha \approx -\rho _\alpha g\varvec{\nu }_\alpha \cdot \nabla _{{\varvec{x}}^\prime }z^\prime =-\rho _\alpha g\varvec{\nu }_\alpha \cdot {\varvec{e}}_3 \quad \mathrm{on}\quad \mathcal {S}_{\alpha _{ij}{\tilde{\beta }}_{ij}}, \end{aligned}$$

which means the microscopic pressure gradient is proportional to the body force in this circumstance. Hence, by additionally assuming $\mathring{\rho }_\alpha \ll \overline{\rho _\alpha }^\alpha$, i.e. $\rho _\alpha \approx \overline{\rho _\alpha }^\alpha =\mathrm{const}$ in the REV, the surface integral on the r.h.s of Eq. (100) reads

104 $$\begin{aligned} \frac{1}{\theta _\alpha U_{0}}\int \limits _{\mathcal {S}_{\alpha _{ij}{\tilde{\beta }}_{ij}}}{(\varvec{\nu }_\alpha \cdot \nabla _{{{\varvec{x}}}^\prime } p_\alpha )\mathring{{\varvec{x}}}^\prime \,\mathrm{{d}}S} \approx -\overline{p_\alpha }^\alpha g{\varvec{e}}_3\cdot \left( \frac{1}{\theta _\alpha U_{0}}\int \limits _{\mathcal {S}_{\alpha _{ij}{\tilde{\beta }}_{ij}}}{\varvec{\nu }_\alpha \otimes \mathring{{\varvec{x}}}^\prime \,\mathrm{{d}}S}\right) . \end{aligned}$$

Using the identity,

105 $$\begin{aligned} {\varvec{I}} \equiv \frac{1}{\theta _\alpha U_{0}}\int \limits _{\mathcal {U}_{0\alpha }}{\nabla \mathring{{\varvec{x}}}^\prime \,\mathrm{{d}}U} = \frac{1}{\theta _\alpha U_{0}}\int \limits _{\mathcal {S}_{\alpha _{ij}{\tilde{\beta }}_{ij}}}{\varvec{\nu }_\alpha \otimes \mathring{{\varvec{x}}}^\prime \,\mathrm{{d}}S} +{\varvec{T}}_\alpha ^*, \end{aligned}$$

Eq. (104) becomes

106 $$\begin{aligned} \frac{1}{\theta _\alpha U_{0}}\int \limits _{\mathcal {S}_{\alpha _{ij}{\tilde{\beta }}_{ij}}}{(\varvec{\nu }_\alpha \cdot \nabla _{{{\varvec{x}}}^\prime } p_\alpha )\mathring{{\varvec{x}}}^\prime \,\mathrm{{d}}S} \approx -\overline{p_\alpha }^\alpha g{\varvec{e}}_3\cdot \left( {\varvec{I}}-{\varvec{T}}_\alpha ^*\right) . \end{aligned}$$

Substituting (106) into Eq. (100) yields

107 $$\begin{aligned} \overline{\nabla _{{\varvec{x}}^\prime }p_\alpha }^\alpha ={\nabla _{{{\varvec{x}}}} \overline{p_\alpha }^\alpha }\cdot {\varvec{T}}_\alpha ^* -\overline{p_\alpha }^\alpha g{\varvec{e}}_3\cdot \left( {\varvec{I}}-{\varvec{T}}_\alpha ^*\right) , \end{aligned}$$

which completes the estimation of the pressure gradient term on the r.h.s of (98). Combining (107) with the gravity term also on the r.h.s of (98),

108 $$\begin{aligned} \theta _\alpha \overline{\rho _\alpha g\nabla _{{\varvec{x}}^\prime }z^\prime }^\alpha =\theta _\alpha \overline{\rho _\alpha }^\alpha g{\varvec{e}}_3, \end{aligned}$$

the two terms are expressed in a concise form,

109 $$\begin{aligned} -\theta _\alpha \overline{\nabla _{{\varvec{x}}^\prime }p_\alpha }^\alpha -\theta _\alpha \overline{\rho _\alpha g\nabla _{{\varvec{x}}^\prime }z^\prime }^\alpha =-\theta _\alpha \left\{ {\nabla _{{{\varvec{x}}}} \overline{p_\alpha }^\alpha } +\overline{p_\alpha }^\alpha g{\varvec{e}}_3\right\} \cdot {\varvec{T}}_\alpha ^*, \end{aligned}$$

which is a function of the macroscopic variable, $\overline{p_\alpha }^\alpha$, and its macroscopic gradient.

#### Averaging the viscous resistance term in Eq. (98)

The third term on the r.h.s of (98) represents the viscous resistance to the flow which naturally may be expressed as a function of the macroscopic velocity, $\overline{{\varvec{V}_\alpha }}^\alpha$, by using a constitutive law of the viscous stress ${\varvec{\tau }}_\alpha$.

As an example, the constitutive law (44) of Newtonian fluid is used here. If the gradients (and deviations) of the material properties, $\mu _\alpha$ and $\lambda _\alpha ^{\prime \prime }$, in the REV can be ignored, Eq. (44) and the averaging rule (55) indicate that

110 $$\begin{aligned} \overline{\nabla _{{\varvec{x}}^\prime }\cdot {\varvec{\tau }_\alpha }}^\alpha =\overline{\mu _\alpha }^\alpha \overline{(\nabla _{{\varvec{x}}^\prime }\cdot \nabla _{{\varvec{x}}^\prime }){\varvec{V}}_\alpha }^\alpha +(\overline{\mu _\alpha }^\alpha +\overline{\lambda _\alpha ^{\prime \prime }}^\alpha )\overline{\nabla _{{\varvec{x}}^\prime }(\nabla _{{\varvec{x}}^\prime }\cdot {\varvec{V}}_\alpha )}^\alpha . \end{aligned}$$

Applying the averaging rules (57) and (58) upon the r.h.s of (110), we obtain111where ${\varvec{q}}_\alpha =\theta _\alpha \overline{\varvec{V}}_\alpha ^\alpha$ is the volume-weighted flux (or specific discharge [5]) of the $\alpha$-phase.

By adopting an additional no-slip condition in the tangential direction on the fluid–solid surface, $\mathcal {S}_{\alpha _{ij}\beta _{ij}^{fs}}$, we have

112 $$\begin{aligned} {\varvec{V}}_\alpha \equiv {\varvec{V}}_s \quad \mathrm{on}\quad \mathcal {S}_{\alpha _{ij}\beta _{ij}^{fs}}, \quad \forall i,j\in \{1,2\}. \end{aligned}$$

Under this assumption and using the relation (72)$_2$, the first surface integral on the r.h.s of (111) becomes113Noting that specifying $\varvec{G}=1$ for Eq. (57) yields  where $\beta (\alpha )$ equals to $\tilde{{\varvec{\beta }}}$ so that

114 $$\begin{aligned} \nabla _{{\varvec{x}}}\theta _\alpha = -\frac{1}{U_0}\int \limits _{\mathcal {S}_{\alpha _{ij}{\tilde{\beta }}_{ij}}}{\varvec{\nu }_\alpha \,\mathrm{{d}}S}. \end{aligned}$$

By using Eq. (114) and an approximation, ${\varvec{V}}_s\big |_{\mathcal {S}_{\alpha _{ij}\beta _{ij}^{fs}}}\approx \overline{{\varvec{V}}_s}^s,$ Eq. (113) is further estimated as115Consequently, the first two terms on the r.h.s of Eq. (111) are expressed together as116where ${\varvec{q}}_{r\alpha }=\theta _\alpha ({\overline{{\varvec{V}}_\alpha }^\alpha -\overline{{\varvec{V}}_s}}^{s} )$ is the macroscopic volume-weighted flux of the $\alpha$-phase relative to the motion of the solid skeleton.

In a similar way, the fourth and fifth terms on the r.h.s of Eq. (111) are estimated together as117With Eqs. (116) and (117) obtained, the remaining work to complete the estimation of the viscous resistance is the estimation of two surface integrals in the third and sixth terms respectively on the r.h.s of (111).

For estimating the third term on the r.h.s of (111), we may calculate that118Noting that $\epsilon \ge 0$ so that ${\varvec{x}}^\prime -\epsilon \varvec{\nu }_\alpha \in \mathcal {U}_{0\alpha }({\varvec{x}},t)$. By assuming $\varvec{\nu }_\alpha \cdot ({\varvec{V}}_\alpha ({\varvec{x}}^\prime )-{\varvec{V}}_\alpha ({\varvec{x}}^\prime -\epsilon \varvec{\nu }_\alpha ))=0$ in an immediate neighbourhood of ${\mathcal {S}_{\alpha _{ij}{\tilde{\beta }}_{ij}}}$ , only the tangential component of ${\varvec{V}}_\alpha ({\varvec{x}}^\prime )-{\varvec{V}}_\alpha ({\varvec{x}}^\prime -\epsilon \varvec{\nu }_\alpha )$, i.e. $({\varvec{V}}_\alpha ({\varvec{x}}^\prime )-{\varvec{V}}_\alpha ({\varvec{x}}^\prime -\epsilon \varvec{\nu }_\alpha ))\cdot ({\varvec{I}}-{\varvec{\nu }}_\alpha \otimes {\varvec{\nu }}_\alpha )$, is remained on the r.h.s of Eq. (118). Hence, taking (72)$_2$ and (112) into account as well, Eq. (118) becomes119Replacing ${\varvec{V}}_s({\varvec{x}}^\prime )$, ${\varvec{V}}_\alpha ({\varvec{x}}^\prime -\epsilon \varvec{\nu }_\alpha )$, and ${\varvec{V}}_\alpha ({\varvec{x}}^\prime )$ in above equation by ${\overline{{\varvec{V}}_s}}^{s}$, $\overline{{\varvec{V}}_\alpha }^\alpha$, and , respectively, and introducing a characteristic length, $\Delta _\alpha$, to replace $\epsilon$, we complete the estimation of the third term on the r.h.s of (111) as120where121$C_\alpha$ and $C_\alpha ^\prime$ are the macroscopic dimensionless shape factors associated with the ${\mathcal {S}}_{\alpha _{ij}\beta _{ij}^{fs}}$ and ${\mathcal {S}}_{\alpha _{ij}\beta _{ij}^{f}}$-surfaces, respectively, on the $\alpha$ side of the surfaces.

For estimating the sixth term on the r.h.s of (111), replacing $\nabla _{{\varvec{x}}^\prime }\cdot {\varvec{V}}_\alpha$ by $\overline{\nabla _{{\varvec{x}}^\prime }\cdot {\varvec{V}}_\alpha }^\alpha$, approximately, and using the relation (114) yield122Specifying $\varvec{G}=\varvec{V}_\alpha$ for Eq. (58) and $\varvec{G}=1$ for Eq. (56), respectively, and summing the two specified equations together lead to123where, again, $\beta (\alpha )$ refers to $\tilde{{\varvec{\beta }}}$. Since ${\mathcal {S}}_{\alpha _{ij}{\tilde{\beta }}_{ij}}$ is a material surface, the surface integral term on the r.h.s of (123) vanishes. Hence, substituting (123) into Eq. (122) completes the estimation of the sixth term on the r.h.s of (111),124By inserting (116), (117), (120), (124) into Eq. (111), we complete the estimation of the viscous resistance,125

#### Averaged momentum balance equation in terms of macroscopic variables

Governed by the last term in mass balance equation (74), the last term on the r.h.s of Eq. (98) represents the momentum exchange between fluids in the intra- and inter-bundle void spaces. This term may be taken as a nonlinear contribution of $\varvec{V}_\alpha$ to (98) and simply estimated as126where $\varvec{u}\approx \overline{{\varvec{V}}_s}^s$ on ${\mathcal {S}}_{\alpha _{ij}\beta _{ij}^{m}}$ since this interface actually is defined by the solid skeleton (see Fig. 3).

Finally, replacing $\overline{{\varvec{V}_\alpha }}^\alpha$ by $\frac{1}{\theta _\alpha }{\varvec{q}_\alpha }$ on the l.h.s of Eq. (98) and substituting (109), (125), (126) into Eq. (98), we obtain the averaged momentum balance equation of the $\alpha$-fluid in terms of the macroscopic variables as127

### First order formulation of macroscopic fluxes

We consider the special case in which (i) the effect of inertia and (ii) the effect of internal friction in the fluid can be neglected in comparison with the viscous resistance force at the fluid–solid and fluid–fluid interfaces, and (iii) the nonlinear term of $\varvec{V}_\alpha$ is negligible as well. In such circumstance, Eq. (127) reduces to128Our aim is to estimate the macroscopic fluxes in a closed form using the above reduced format of momentum balance equation. For this purpose, the surface integral of microscopic velocity, , in Eq. (128) need to be expressed as a linear function of the macroscopic fluxes.

#### Averaged fluid velocity on the ${\mathcal {S}}_{\alpha _{ij}\beta _{ij}^u}$-surface

The expression of  depends on the force balance on the ${\mathcal {S}}_{\alpha _{ij}\beta _{ij}^u}$-interface between two immiscible fluids. Noting that the force balance equation (81) on the $\mathcal {S}_{{\alpha _{ij}}\beta _{ij}^c}$-interface can be equivalently re-formulated to the $\mathcal {S}_{{\alpha _{ij}}\beta _{ij}^u}$-interface as

129 $$\begin{aligned} \varvec{\nu }_{\alpha _{ij}}\cdot [{{\varvec{\sigma }}}]_{\alpha _{ij},\beta _{ij}^u} =\mathrm{sign}(\beta _{ij}^u)\frac{2}{r^*}\gamma _{\alpha \beta ^u}\varvec{\nu }_{\alpha _{ij}} -|\nabla _{{{\varvec{x}}}^\prime }\gamma _{\alpha \beta ^c}|{\varvec{t}}, \end{aligned}$$

which shows that the tangential force balance equation on a $\mathcal {S}_{{\alpha _{ij}}\beta _{ij}^u}$-interface has the same form as (83). Thus, by using the constitutive law (44) and kinematical relation, $\varvec{\nu }_\alpha =-\varvec{\nu }_{\beta _{ij}^u}$, Eq. (83) indicates

130 $$\begin{aligned}&\mu _\alpha ((\varvec{\nu }_\alpha \cdot \nabla _{{{\varvec{x}}}^\prime }\varvec{V}_\alpha )\cdot {\varvec{t}} +\varvec{\nu }_\alpha \cdot (\varvec{V}_\alpha \nabla _{{{\varvec{x}}}^\prime }\cdot {\varvec{t}})) +\mu _{\beta _{ij}^u}((\varvec{\nu }_{\beta _{ij}^u}\cdot \nabla _{{{\varvec{x}}}^\prime }\varvec{V}_{\beta _{ij}^u})\cdot {\varvec{t}} +\varvec{\nu }_{\beta _{ij}^u}\cdot (\varvec{V}_{\beta _{ij}^u}\nabla _{{{\varvec{x}}}^\prime }\cdot {\varvec{t}}))\nonumber \\&\quad = -\nabla _{{{\varvec{x}}}^\prime }\gamma _{\alpha \beta ^u}\cdot {\varvec{t}} \quad \mathrm{on}\quad \mathcal {S}_{\alpha _{ij}\beta _{ij}^{u}}, \quad \forall i,j\in \{1,2\}. \end{aligned}$$

By assuming that the dominant fluid velocity variation in the close vicinity of the ${\mathcal {S}}_{\alpha _{ij}\beta _{ij}^u}$-interface is the variation of the tangential velocity along the normal direction $\varvec{\nu }_\alpha$, Eq. (130) reduces to

131 $$\begin{aligned} \mu _\alpha \varvec{\nu }_\alpha \cdot \nabla _{{{\varvec{x}}}^\prime }\varvec{V}_\alpha +\mu _{\beta _{ij}^u}\varvec{\nu }_{\beta _{ij}^u}\cdot \nabla _{{{\varvec{x}}}^\prime }\varvec{V}_{\beta _{ij}^u} \approx -\nabla _{{{\varvec{x}}}^\prime }\gamma _{\alpha \beta ^u}. \end{aligned}$$

Taking average of Eq. (131) over the $\mathcal {S}_{{\alpha _{ij}}\beta _{ij}^u}$-interface within the REV, we come to an estimation,132Proceeding in a way similar to that used in Eqs. (118)–(120), Eq. (132) yields133Adopting the additional no-slip condition on the material surface ${\mathcal {S}}_{\alpha _{ij}\beta _{ij}^u}$ leads to

134 $$\begin{aligned} {\varvec{V}}_\alpha \equiv {\varvec{V}}_{\beta _{ij}^u} \quad \mathrm{on}~{\mathcal {S}}_{\alpha _{ij}\beta _{ij}^u}. \end{aligned}$$

Therefore, we can work out  from (133) as135

#### Closed form solution of fluxes

Inserting (135) into Eq. (128), we obtain the reduced format of momentum balance equation of the $\alpha$-fluid as a linear equation of the macroscopic fluxes,

136 $$\begin{aligned} {{\varvec{q}}_{r\alpha }\cdot {\varvec{A}}_{\alpha \alpha }}+{{\varvec{q}}_{r\beta _{ij}^u}\cdot {\varvec{A}}_{\alpha \beta ^u}}={\varvec{b}}_\alpha , \end{aligned}$$

where137138in which

139 $$\begin{aligned} {\varvec{k}}_\alpha =\frac{\Delta _\alpha }{C_\alpha }\frac{\theta _\alpha ^2}{{\bar{S}}_{\alpha _{ij}\beta _{ij}^{fs}}}{{\varvec{T}}_\alpha ^*}\cdot ({{\varvec{\alpha }}^{\alpha _{ij}\beta _{ij}^{fs}}})^{-1} \quad \mathrm{and}\quad a^{\beta ^u\alpha }= \frac{ {\bar{S}}_{\alpha _{ij}\beta _{ij}^u} }{ \frac{\Delta _{\beta _{ij}^u}}{{\overline{\mu _{\beta _{ij}^u}}}^{\beta _{ij}^u}C_{\beta _{ij}^u}^\prime } +\frac{\Delta _\alpha }{{\overline{\mu _\alpha }}^\alpha C_\alpha ^\prime } }. \end{aligned}$$

By interchanging $\alpha$ with $\beta _{ij}^u$, we have the other equation of the phase pair, $\alpha -\beta _{ij}^u$,

140 $$\begin{aligned} {{\varvec{q}}_{r\beta _{ij}^u}\cdot {\varvec{A}}_{\beta ^u\beta ^u}}+{{\varvec{q}}_{r\alpha }\cdot {\varvec{A}}_{\beta ^u\alpha }}={\varvec{b}}_{\beta ^u}, \end{aligned}$$

where

141 $$\begin{aligned} {\varvec{A}}_{\beta ^u\beta ^u}= \overline{\mu _{\beta _{ij}^u}}^{\beta _{ij}^u}\theta _{\beta _{ij}^u}{\varvec{k}}_{\beta _{ij}^u}^{-1}{{\varvec{T}}_{\alpha _{IJ}}} + \frac{1}{\theta _{\beta _{ij}^u}}a^{\beta ^u\alpha } {\varvec{\alpha }}^{\alpha _{ij}\beta _{ij}^u},\quad {\varvec{A}}_{\beta ^u\alpha }= \frac{1}{\theta _\alpha }a^{\beta ^u\alpha } {\varvec{\alpha }}^{\alpha _{ij}\beta _{ij}^u}, \end{aligned}$$

and142in which

143 $$\begin{aligned} {\varvec{k}}_{\beta _{ij}^u} =\frac{\Delta _{\beta _{ij}^u}}{C_{\beta _{ij}^u}}\frac{\theta _{\beta _{ij}^u}^2}{S_{\alpha _{IJ}\beta _{IJ}^{fs}}}{{\varvec{T}}_{\alpha _{IJ}}^*}\cdot ({{\varvec{\alpha }}^{\alpha _{IJ}\beta _{IJ}^{fs}}})^{-1}. \end{aligned}$$

Noting that we use a transfer, $\alpha _{IJ}=|\beta _{ij}^u|$, where, given the indexes *i*, *j*, it can be proven that there exist $I,J\in \{1,2\}$ satisfy such a transfer.

Solving the set of linear Eqs. (136) and (140), we obtain the macroscopic fluxes of the phase pair, $\alpha -\beta _{ij}^u$,

144 $$\begin{aligned} \begin{Bmatrix} {\varvec{q}}_{r\alpha }\\ {\varvec{q}}_{r{\beta _{ij}^u}}\\ \end{Bmatrix} =\begin{Bmatrix} \left( {\varvec{A}}_\alpha + \frac{1}{\theta _{\beta _{ij}^u}}\hat{{\varvec{\alpha }}}{\varvec{A}}_{\beta ^u}^{-1}{\varvec{A}}_\alpha +\frac{1}{\theta _\alpha }\hat{{\varvec{\alpha }}}\right) ^{-1} \left\{ ({\varvec{I}}+ \frac{1}{\theta _{\beta _{ij}^u}}\hat{{\varvec{\alpha }}}{\varvec{A}}_{\beta ^u}^{-1}){\varvec{b}}_\alpha ^\mathrm{{T}} -\frac{1}{\theta _{\beta _{ij}^u}}\hat{{\varvec{\alpha }}}{\varvec{A}}_{\beta ^u}^{-1}{\varvec{b}}_{\beta ^u}^\mathrm{{T}}\right\} \\ \left( {\varvec{A}}_{\beta ^u}+ \frac{1}{\theta _\alpha }\hat{{\varvec{\alpha }}}{\varvec{A}}_\alpha ^{-1}{\varvec{A}}_{\beta ^u}+\frac{1}{\theta _{\beta _{ij}^u}}\hat{{\varvec{\alpha }}}\right) ^{-1} \left\{ ({\varvec{I}}+ \frac{1}{\theta _\alpha }\hat{{\varvec{\alpha }}}{\varvec{A}}_\alpha ^{-1}){\varvec{b}}_{\beta ^u}^\mathrm{{T}} -\frac{1}{\theta _\alpha }\hat{{\varvec{\alpha }}}{\varvec{A}}_\alpha ^{-1}{\varvec{b}}_\alpha ^\mathrm{T}\right\} \\ \end{Bmatrix}, \end{aligned}$$

where

145 $$\begin{aligned} {\varvec{A}}_\alpha ^\mathrm{{T}}=\overline{\mu _\alpha }^\alpha \theta _\alpha {\varvec{k}}_\alpha ^{-1}{{\varvec{T}}_\alpha ^*}, \quad {\varvec{A}}_{\beta ^u}^\mathrm{{T}}=\overline{\mu _{\beta _{ij}^u}}^{\beta _{ij}^u}\theta _{\beta _{ij}^u}{\varvec{k}}_{\beta _{ij}^u}^{-1}{{\varvec{T}}_{\alpha _{IJ}}^*}, \quad \hat{{\varvec{\alpha }}}^\mathrm{{T}}=a^{{\beta ^u}\alpha }{\varvec{\alpha }}^{\alpha _{ij}{\beta _{ij}^u}}. \end{aligned}$$

By specifying $\alpha =wb,wf$ in (144), we can calculate two pairs, $$\begin{Bmatrix} {\varvec{q}}_{rwb}\\ {\varvec{q}}_{rnb}\\ \end{Bmatrix}$$ and $$\begin{Bmatrix} {\varvec{q}}_{rwf}\\ {\varvec{q}}_{rnf}\\ \end{Bmatrix}$$, respectively, which completes the estimation of the macroscopic fluxes of the dual scale multiphase flow in a closed form.

It is interesting to observe the driving forces of the macroscopic flux of an $\alpha$-phase shown in Eq. (144). First, the $\alpha$-phase’s own macroscopic pressure gradient plays a role as described by Darcy’s law. Secondly, there is an explicit coupling between two neighbouring immiscible phases. The forces due to pressure gradient and gravity in one fluid cause the motion in the other one, e.g. ${\varvec{b}}_{\beta ^u}$ contributing to ${\varvec{q}}_{r\alpha }$, due to the momentum exchange at their interface. Thirdly, the inhomogeneity in the surface tension, , acts as an additional driving force. Finally but implicitly, the interaction between the intra- and inter-bundle flows which is represented in the mass balance equation (74) and tortuosity ${\varvec{T}}_\alpha ^*$, is computed on the $\mathcal {S}_{\alpha _{ij}{\tilde{\alpha }}_{ij}}$-interface.

### Quadratic formulation of macroscopic fluxes

The other reduced format of momentum balance derived from Eq. (127) is146By comparison to Eq. (128) which leads to the closed form solution (144), Eq. (146) takes into account a nonlinear term of the velocities which explicitly represents the interaction between the intra- and inter-bundle flows.

We use two approximations,

147 $$\begin{aligned} {\varvec{V}_\alpha }-\overline{{\varvec{V}}_s}^s\approx \overline{\varvec{V}_\alpha }^\alpha -\overline{{\varvec{V}}_s}^s \end{aligned}$$

and

148 $$\begin{aligned} {\varvec{V}_\alpha }-\overline{\varvec{V}_\alpha }^\alpha \approx \frac{1}{\theta _{\alpha _{ij}}+\theta _{\beta _{ij}^m}}(\theta _{\alpha _{ij}}\overline{\varvec{V}_\alpha }^\alpha +\theta _{\beta _{ij}^m}\overline{\varvec{V}_{\beta _{ij}^m}}^{\beta _{ij}^m})-\overline{\varvec{V}_\alpha }^\alpha = -\frac{\theta _{\beta _{ij}^m}}{\theta _{\alpha _{ij}}+\theta _{\beta _{ij}^m}} (\overline{\varvec{V}_\alpha }^\alpha -\overline{\varvec{V}_{\beta _{ij}^m}}^{\beta _{ij}^m}). \end{aligned}$$

Thus the nonlinear term in Eq. (146) becomes149where150Substituting (149) into Eq. (146) and using Eq. (136), we obtain the quadratic formulation of the macroscopic fluxes,

151 $$\begin{aligned} {{\varvec{q}}_{r\alpha }\cdot {\varvec{A}}_{\alpha \alpha }}+{{\varvec{q}}_{r\beta _{ij}^u}\cdot {\varvec{A}}_{\alpha \beta ^u}} -{\varvec{d}}_{\alpha \beta ^m} \cdot {\varvec{q}}_{r\alpha } \otimes \left( \frac{\theta _{\beta _{ij}^m}}{\theta _{\alpha _{ij}}}{\varvec{q}}_{r\alpha } -{\varvec{q}}_{r\beta _{ij}^m}\right) ={\varvec{b}}_\alpha . \end{aligned}$$

For computing its solution, Eq. (151) may be re-arranged as a nonlinear equation of a quadratic function $\mathcal {F}$,

152 $$\begin{aligned} \mathcal {F}({\varvec{q}}_{r\alpha },{\varvec{q}}_{r\beta _{ij}^u},{\varvec{q}}_{r\beta _{ij}^m})={{\varvec{q}}_{r\alpha }\cdot {\varvec{A}}_{\alpha \alpha }}+{{\varvec{q}}_{r\beta _{ij}^u}\cdot {\varvec{A}}_{\alpha \beta ^u}} -{\varvec{d}}_{\alpha \beta ^m} \cdot {\varvec{q}}_{r\alpha } \otimes \left( \frac{\theta _{\beta _{ij}^m}}{\theta _{\alpha _{ij}}}{\varvec{q}}_{r\alpha } -{\varvec{q}}_{r\beta _{ij}^m}\right) -{\varvec{b}}_\alpha =0. \end{aligned}$$

The linearised incremental form of above equation is

153 $$\begin{aligned} \Delta {\varvec{q}}_{r\alpha }\cdot {\varvec{A}}_{\alpha \alpha }^\prime +\Delta {\varvec{q}}_{r\beta _{ij}^u}\cdot {\varvec{A}}_{\alpha \beta ^u} +\Delta {\varvec{q}}_{r\beta _{ij}^m}\cdot {\varvec{A}}_{\alpha \beta ^m}^\prime -\mathcal {F}({\varvec{q}}_{r\alpha },{\varvec{q}}_{r\beta _{ij}^u},{\varvec{q}}_{r\beta _{ij}^m})=0, \end{aligned}$$

where $\Delta$ is the symbol of increment,

154 $$\begin{aligned} {\varvec{A}}_{\alpha \alpha }^\prime ={\varvec{A}}_{\alpha \alpha } -{\varvec{d}}_{\alpha \beta ^m} \otimes \left( \frac{\theta _{\beta _{ij}^m}}{\theta _{\alpha _{ij}}}{\varvec{q}}_{r\alpha } -{\varvec{q}}_{r\beta _{ij}^m}\right) - \frac{\theta _{\beta _{ij}^m}}{\theta _{\alpha _{ij}}}({\varvec{d}}_{\alpha \beta ^m}\cdot {\varvec{q}}_{r\alpha }){\varvec{I}}, \end{aligned}$$

and

155 $$\begin{aligned} {\varvec{A}}_{\alpha \beta ^m}^\prime 
=({\varvec{d}}_{\alpha \beta ^m}\cdot {\varvec{q}}_{r\alpha }){\varvec{I}}. \end{aligned}$$

The incremental equation of the whole dual scale multiphase flow system can be obtained by specifying Eq. (153) for each phase and assembling the equations of all fluid phases together

156 $$\begin{aligned} \begin{bmatrix} {\varvec{A}}_{wbwb}^{\prime \mathrm{{T}}} &{} {\varvec{A}}_{wbnb}^\mathrm{{T}} &{} {\varvec{A}}_{wbwf}^{\prime \mathrm{{T}}} &{} {\varvec{0}}\\ {\varvec{A}}_{nbwb}^\mathrm{{T}} &{}{\varvec{A}}_{nbnb}^{\prime \mathrm{{T}}} &{} {\varvec{0}} &{} {\varvec{A}}_{nbnf}^{\prime \mathrm{{T}}} \\ {\varvec{A}}_{wfwb}^{\prime \mathrm{{T}}} &{} {\varvec{0}} &{} {\varvec{A}}_{wfwf}^{\prime \mathrm{{T}}} &{} {\varvec{A}}_{wfnf}^\mathrm{{T}} \\ {\varvec{0}} &{} {\varvec{A}}_{nfnb}^{\prime \mathrm{T}} &{} {\varvec{A}}_{nfwf}^\mathrm{{T}} &{} {\varvec{A}}_{nfnf}^{\prime \mathrm{{T}}} \\ \end{bmatrix} \begin{Bmatrix} \Delta {\varvec{q}}_{rwb}\\ \Delta {\varvec{q}}_{rnb}\\ \Delta {\varvec{q}}_{rwf}\\ \Delta {\varvec{q}}_{rnf}\\ \end{Bmatrix} = \begin{Bmatrix} \mathcal {F}({\varvec{q}}_{rwb},{\varvec{q}}_{rnb},{\varvec{q}}_{rwf})\\ \mathcal {F}({\varvec{q}}_{rnb},{\varvec{q}}_{rwb},{\varvec{q}}_{rnf})\\ \mathcal {F}({\varvec{q}}_{rwf},{\varvec{q}}_{rnf},{\varvec{q}}_{rwb})\\ \mathcal {F}({\varvec{q}}_{rnf},{\varvec{q}}_{rwf},{\varvec{q}}_{rnb})\\ \end{Bmatrix}. \end{aligned}$$

The above equation explicitly reflects the coupling effects between the different phases.

### Brinkman-type formulation of macroscopic fluxes

If the internal friction term, $\overline{\mu _\alpha }^\alpha \nabla _{{\varvec{x}}}\cdot \nabla _{{\varvec{x}}}{{\varvec{q}}_{r\alpha }}$, is considered and added to Eq. (128), we have the third reduced format of momentum balance derived from (127) as157By using the expression (136), Eq. (157) becomes

158 $$\begin{aligned} {{\varvec{q}}_{r\alpha }\cdot {\varvec{A}}_{\alpha \alpha }}+{{\varvec{q}}_{r\beta _{ij}^u}\cdot {\varvec{A}}_{\alpha \beta ^u}}={\varvec{b}}_\alpha -\overline{\mu _\alpha }^\alpha \nabla _{{\varvec{x}}}\cdot \nabla _{{\varvec{x}}}{{\varvec{q}}_{r\alpha }}. \end{aligned}$$

It is difficult to obtain a closed form solution of the above Brinkman-type equation for an REV domain. On the other hand, from the point of view of FEA, one may take Eq. (158) as a point-wise constitutive equation to be computed at the Gaussian points of each element. We shall discuss a suitable numerical method for computing (158) elsewhere.

## Conclusions

Vacuum assisted Resin Infusion is an important manufacturing method for composite materials. However, it is a great challenge to design the infusion system and infusion process based on an effective prediction of the resin flow in a dual scale fibre-reinforced composite preform. At the component scale, it may need the costly and time-consuming small scale and full scale trial and error tests to mitigate the dry domains due to the effects of flow paths. At material scale, the air may flow and be trapped at the intra- and inter-bundle void spaces, which results in the defects after curing and may need the expensive nondestructive defect detection to evaluate the risk. Moreover, the fluid–solid interaction, which is a transient process due to the change of the resin pressure during an infusion process, makes the material property, e.g. permeability, process-dependent and thus adds further difficulty to the process modelling.

One of the essential challenges in finite element modelling of RI is the constitutive model capable to capture and feasible to predict the above key features of the resin infusion. Macroscopic constitutive modelling based on thermodynamic constraints and material symmetry, which generally leads to a phenomenological Darcy’s law, may lack the high-fidelity to accurately depict the phenomena in dual porosity fibre-reinforced media and accordingly is not able to provide the effective prediction to support the design of infusion system and process, especially for large-scale composite components.

By taking the same fluid in the intra- and inter-bundle void spaces as the different phases, the present study propose a mathematical framework aiming at effectively clarifying and predicting the dual scale multiphase flow phenomena in the resin infusion. First, the fundamental physics laws of each phase are proposed in the finite strain format to fully understand the system at microstructure level. The microscopic mass balance and linear momentum balance equations play the role to understand the motion of phases while the energy balance and entropy balance lead to the constitutive laws of each phase. Secondly, a symbolic system, denoted phase matrices, is proposed to clarify the topological relationship between different phases. Using this symbolic system, the averaged mass balance and linear momentum balance equations are obtained as the governing equations of the macroscopic modelling. And the macroscopic finite strain constitutive laws of stresses can be obtained by the homogenisation and development of the microscopic counterparts. This is important to address the fluid–solid interaction. Finally, the fluid fluxes are directly derived from the averaged momentum balance equations, which is aiming to clarify the mechanisms of the multiphase flow in a dual scale medium and sheds new light on a constitutive model suitable for FEA. Analytical analysis and finite element modelling based on the present study will be discussed in future work for further validation and application.

In brief, the present study serves to clarify the microscopic mechanisms and to bridge the gap between microscopic observation and macroscopic modelling of the resin infusion process. The proposed method potentially may be helpful to the research in other areas involving multiphase flows in dual scale fibre-reinforced porous media.

## Appendix A

Let’s consider the trace format of Gauss’s theorem,

A.1 $$\begin{aligned} \int \limits _{\mathcal {S}}{\varvec{\nu }\cdot \varvec{G}\,\mathrm{{d}}S} =\int \limits _{\mathcal {U}}{\nabla \cdot \varvec{G}\,\mathrm{{d}}U}, \end{aligned}$$

where $\varvec{\nu }$ is the outer normal vector and ${\varvec{G}}$ is any tensor, $\mathcal {U}$ is a domain bounded by a surface $\mathcal {S}$.

By specifying ${\varvec{G}}=b\nabla {\varvec{a}}$ for a scalar *b* and vector ${\varvec{a}}$, we obtain

A.2 $$\begin{aligned} \int \limits _{\mathcal {S}}{b\varvec{\nu }\cdot \nabla {\varvec{a}}\; \mathrm{{d}}S} =\int \limits _{\mathcal {U}}{(\nabla b)\cdot \nabla {\varvec{a}})\,\mathrm{{d}}U} +\int \limits _{\mathcal {U}}{b\nabla \cdot \nabla {\varvec{a}}\,\mathrm{{d}}U}. \end{aligned}$$

Likewise, let ${\varvec{G}}=(\nabla b)\otimes {\varvec{a}}$, we have

A.3 $$\begin{aligned} \int \limits _{\mathcal {S}}{\varvec{\nu }\cdot (\nabla b)\otimes {\varvec{a}}\,\mathrm{{d}}S} =\int \limits _{\mathcal {U}}{(\nabla \cdot \nabla b){\varvec{a}}\,\mathrm{{d}}U} +\int \limits _{\mathcal {U}}{(\nabla b)\cdot \nabla {\varvec{a}}\,\mathrm{{d}}U}. \end{aligned}$$

By subtracting Eq. (A.3) from Eq. (A.2), we obtain Green’s (vector) theorem

A.4 $$\begin{aligned} \int \limits _{\mathcal {S}}{\varvec{\nu }\cdot (b\nabla {\varvec{a}} -(\nabla b)\otimes {\varvec{a}})\mathrm{{d}}S} =\int \limits _{\mathcal {U}}{b\nabla \cdot \nabla {\varvec{a}}-(\nabla \cdot \nabla b){\varvec{a}}\,\mathrm{{d}}U}. \end{aligned}$$

Specifying ${\varvec{a}}=\mathring{{\varvec{x}}}^\prime$, $b=G$, and $\mathcal {U}=\mathcal {U}_{0\alpha }$, Eq. (A.4) reads

A.5 $$\begin{aligned} \int \limits _{\mathcal {S}_{0\alpha }}{\varvec{\nu }\cdot (G\nabla _{{{\varvec{x}}}^\prime } \mathring{{\varvec{x}}}^\prime -(\nabla _{{{\varvec{x}}}^\prime } G)\otimes \mathring{{\varvec{x}}}^\prime )\,\mathrm{{d}}S} =\int \limits _{\mathcal {U}_{0\alpha }}{G\nabla _{{{\varvec{x}}}^\prime }^2 \mathring{{\varvec{x}}}^\prime -(\nabla _{{{\varvec{x}}}^\prime }^2 G)\mathring{{\varvec{x}}}^\prime \,\mathrm{{d}}U}. \end{aligned}$$

Since $\nabla _{{{\varvec{x}}}^\prime } \overline{\varvec{x}^\prime }={\varvec{0}}$ and $\nabla _{{{\varvec{x}}}^\prime } {{\varvec{x}}}^\prime ={\varvec{I}}$ within an REV, we have

$$\begin{aligned}\nabla _{{{\varvec{x}}}^\prime } \mathring{{\varvec{x}}}^\prime ={\varvec{I}} \quad \mathrm{and}\quad \nabla _{{{\varvec{x}}}^\prime }^2 \mathring{{\varvec{x}}}^\prime ={\varvec{0}}.\end{aligned}$$

Therefore, Eq. (A.5) reduces to

A.6 $$\begin{aligned} \int \limits _{\mathcal {S}_{0\alpha }}{G\varvec{\nu }\,\mathrm{{d}}S} =\int \limits _{\mathcal {S}_{0\alpha }}{(\varvec{\nu }\cdot \nabla _{{{\varvec{x}}}^\prime } G)\mathring{{\varvec{x}}}^\prime \,\mathrm{{d}}S} -\int \limits _{\mathcal {U}_{0\alpha }}{ (\nabla _{{{\varvec{x}}}^\prime }^2 G)\mathring{{\varvec{x}}}^\prime \,\mathrm{{d}}U}. \end{aligned}$$

Let’s consider a scalar function, $G({{\varvec{x}}}^\prime ,t)$, satisfying two conditions,

A.7 $$\begin{aligned} \nabla _{{{\varvec{x}}}^\prime }^2 G=0 \quad \mathrm{within}\quad \mathcal {U}_{0\alpha } \end{aligned}$$

and

A.8 $$\begin{aligned} \int \limits _{\mathcal {S}_{\alpha _{ij}{\tilde{\alpha }}_{ij}}}{(\varvec{\nu }\cdot \nabla _{{{\varvec{x}}}^\prime } G)\mathring{{\varvec{x}}}^\prime \,\mathrm{{d}}S} \approx {\nabla _{{{\varvec{x}}}} \overline{G}^\alpha } \cdot \int \limits _{\mathcal {S}_{\alpha _{ij}{\tilde{\alpha }}_{ij}}}{\varvec{\nu } \otimes \mathring{{\varvec{x}}}^\prime \,\mathrm{{d}}S}, \end{aligned}$$

where we use the approximation,

$$\begin{aligned} \frac{1}{S_{\alpha _{ij}{\tilde{\alpha }}_{ij}}} \int \limits _{\mathcal {S}_{\alpha _{ij}{\tilde{\alpha }}_{ij}}}{( \nabla _{{{\varvec{x}}}^\prime } G)\,\mathrm{{d}}S} \approx {\nabla _{{{\varvec{x}}}} \overline{G}^\alpha }. \end{aligned}$$

Substituting Eqs. (A.7) and (A.8) into its r.h.s, Eq. (A.6) reduces to

A.9 $$\begin{aligned} \int \limits _{\mathcal {S}_{0\alpha }}{G\varvec{\nu }\,\mathrm{{d}}S} =\int \limits _{\mathcal {S}_{\alpha _{ij}{\tilde{\beta }}_{ij}}}{(\varvec{\nu }\cdot \nabla _{{{\varvec{x}}}^\prime } G)\mathring{{\varvec{x}}}^\prime \,\mathrm{{d}}S} +{\nabla _{{{\varvec{x}}}} \overline{G}^\alpha } \cdot \int \limits _{\mathcal {S}_{\alpha _{ij}{\tilde{\alpha }}_{ij}}}{\varvec{\nu } \otimes \mathring{{\varvec{x}}}^\prime \,\mathrm{{d}}S}. \end{aligned}$$

Applying Gauss’s theorem to the l.h.s of Eq. (A.9),

A.10 $$\begin{aligned} \int \limits _{\mathcal {S}_{0\alpha }}{G\varvec{\nu }\,\mathrm{{d}}S} =\int \limits _{\mathcal {U}_{0\alpha }}{\nabla _{{\varvec{x}}^\prime } G\,\mathrm{{d}}U} =U_{0\alpha }\overline{\nabla _{{\varvec{x}}^\prime }G}^{\alpha }, \end{aligned}$$

Equation (A.9) becomes

A.11 $$\begin{aligned} \overline{\nabla _{{\varvec{x}}^\prime }G^{\alpha }} ={\nabla _{{{\varvec{x}}}} \overline{G}^\alpha }\cdot {\varvec{T}}_\alpha ^* +\frac{1}{U_{0\alpha }}\int \limits _{\mathcal {S}_{\alpha _{ij}{\tilde{\beta }}_{ij}}}{(\varvec{\nu }\cdot \nabla _{{{\varvec{x}}}^\prime } G)\mathring{{\varvec{x}}}^\prime \,\mathrm{{d}}S}, \end{aligned}$$

where

A.12 $$\begin{aligned} {\varvec{T}}_\alpha ^* =\frac{1}{U_{0\alpha }}\int \limits _{\mathcal {S}_{\alpha _{ij}{\tilde{\alpha }}_{ij}}}{\varvec{\nu } \otimes \mathring{{\varvec{x}}}^\prime \,\mathrm{{d}}S}. \end{aligned}$$
